# An Expanded Multilocus Sequence Typing Scheme for *Propionibacterium acnes*: Investigation of ‘Pathogenic’, ‘Commensal’ and Antibiotic Resistant Strains

**DOI:** 10.1371/journal.pone.0041480

**Published:** 2012-07-30

**Authors:** Andrew McDowell, Emma Barnard, István Nagy, Anna Gao, Shuta Tomida, Huiying Li, Anne Eady, Jonathan Cove, Carl E. Nord, Sheila Patrick

**Affiliations:** 1 Centre for Infection and Immunity, School of Medicine, Dentistry and Biomedical Sciences, Queen’s University, Belfast, United Kingdom; 2 Institute of Biochemistry, Biological Research Centre of the Hungarian Academy of Sciences, Szeged, Hungary; 3 School of Life Sciences, University of Warwick, Coventry, United Kingdom; 4 Department of Molecular and Medical Pharmacology, Crump Institute for Molecular Imaging, University of California Los Angeles, Los Angeles, California, United States of America; 5 Department of Dermatology, Harrogate and District NHS Foundation Trust, Harrogate, United Kingdom; 6 Faculty of Biological Sciences, University of Leeds, Leeds, United Kingdom; 7 Department of Laboratory Medicine, Karolinska University Hospital Huddinge, Karolinska Institute, Stockholm, Sweden; The University of Hong Kong, Hong Kong

## Abstract

The Gram-positive bacterium *Propionibacterium acnes* is a member of the normal human skin microbiota and is associated with various infections and clinical conditions. There is tentative evidence to suggest that certain lineages may be associated with disease and others with health. We recently described a multilocus sequence typing scheme (MLST) for *P. acnes* based on seven housekeeping genes (http://pubmlst.org/pacnes). We now describe an expanded eight gene version based on six housekeeping genes and two ‘putative virulence’ genes (eMLST) that provides improved high resolution typing (91eSTs from 285 isolates), and generates phylogenies congruent with those based on whole genome analysis. When compared with the nine gene MLST scheme developed at the University of Bath, UK, and utilised by researchers at Aarhus University, Denmark, the eMLST method offers greater resolution. Using the scheme, we examined 208 isolates from disparate clinical sources, and 77 isolates from healthy skin. Acne was predominately associated with type IA_1_ clonal complexes CC1, CC3 and CC4; with eST1 and eST3 lineages being highly represented. In contrast, type IA_2_ strains were recovered at a rate similar to type IB and II organisms. Ophthalmic infections were predominately associated with type IA_1_ and IA_2_ strains, while type IB and II were more frequently recovered from soft tissue and retrieved medical devices. Strains with rRNA mutations conferring resistance to antibiotics used in acne treatment were dominated by eST3, with some evidence for intercontinental spread. In contrast, despite its high association with acne, only a small number of resistant CC1 eSTs were identified. A number of eSTs were only recovered from healthy skin, particularly eSTs representing CC72 (type II) and CC77 (type III). Collectively our data lends support to the view that pathogenic versus truly commensal lineages of *P. acnes* may exist. This is likely to have important therapeutic and diagnostic implications.

## Introduction

*Propionibacterium acnes* is a Gram-positive aerotolerant anaerobe that forms part of the normal resident microbiota of the skin, oral cavity and the gastrointestinal and genito-urinary tracts [1]. It is an opportunistic pathogen and has been linked to a wide range of infections and conditions, including acne vulgaris [2], medical device [3]–[5], dental [6] and ophthalmic [7] infections, as well as synovitis-acne-pustulosis-hyperostosis-osteitis (SAPHO) syndrome [8], sarcoidosis [9], [10] and prostate cancer [11]. Previously, we demonstrated that *P. acnes* comprises four highly distinct evolutionary lineages, known as type IA, IB, II and III that display differences in inflammatory properties, production of virulence determinants and association with various conditions [12]–[16].

To build and expand on these earlier phylogenetic and epidemiological studies, we recently described a multilocus sequence typing (MLST) scheme for *P. acnes* which was validated against Random Amplification of Polymorphic DNA (RAPD) and antibody typing methods [17]. This scheme and its corresponding database, which were established in 2005 and announced on http://pubmlst.org/, is based on partial nucleotide sequences from seven core housekeeping genes (3135 bp). Upon concatenated gene sequence analysis, this original MLST resolves isolates into specific sequence types (STs) within the phylogenetic divisions IA, IB, II and III, and also divides strains from the large type IA clade into two highly distinct groups, designated types IA_1_ and IA_2,_ which is supported by phylogenomic analysis of multi-housekeeping gene datasets compiled from completed and currently ongoing *P. acnes* whole genome sequencing projects [17]–[19]. We previously used this MLST scheme to highlight the association of acne and ophthalmic infections with STs from the type IA division [17] and since then, the method has also been utilised by other research groups [20]. A recent study by Kilian et al. [18] demonstrated that MLST analysis with nine housekeeping genes affords additional discriminatory power for the identification of particular *P. acnes* clones and lineages (hereafter called the Aarhus scheme) when compared to our previously described method based on fewer loci. Their protocol utilises the genes and their corresponding primer sequences that were originally developed for the typing of *P. acnes* at the University of Bath, UK (O’Hanlon et al.; http://www.mlst.net/comingsoon/pacnes.asp), only one of which (*recA*) is shared between the two schemes. The increased window of discrimination described with the use of additional genes primarily relates to the type IA_1_ clade and, in particular, isolates previously classified as genotype ST6 [17]; although the clinical relevance of these additional subtypes has not been clearly established. Against this background, we now report an updated and expanded MLST (eMLST) method in which one housekeeping gene (*recA*) has been removed, and the complete sequences from two ‘putative virulence’ genes, namely a haemolysin (*tly*) and a Christie-Atkins-Munch-Petersen (CAMP) factor homologue (*camp 2*) added. This eight-gene based version (4253 bp) demonstrates greater levels of resolution compared to the MLST method originally described by Lomholt and Kilian [21] when analysed against a panel of 86 *P. acnes* isolates. We describe application of the expanded scheme to a large collection of isolates recovered from diverse clinical samples (n = 208), as well as healthy skin (n = 77), to investigate whether ‘pathogenic’ versus truly ‘commensal’ lineages may exist. We also describe the first population genetic analysis of isolates with rRNA mutations conferring resistance to tetracyclines, erythromycin and clindamycin, antibiotics commonly used to treat acne patients.

## Results and Discussion

### Allelic Variation in Virulence and Surface Antigen Genes

To further develop our MLST scheme for enhanced discrimination of isolates, primarily those from the type I clade, we examined a range of genes that encode ‘putative virulence’ factors. Such genes, especially those encoding cell surface-associated antigens, are being increasing utilised in MLST schemes as they may be under positive selection, which can result in enhanced diversity and discriminatory power, and can also provide information on the evolution of virulence [22], [23].

We identified a number of candidate genes (n = 11) from the literature that encode ‘putative virulence’ factors and cell surface antigens in *P. acnes* and assessed their suitability for our eMLST scheme based on their locations within the genome and levels of diversity (Table 1). Genes selected included two putative cell invasion-associated proteins (*pamce*; *pap60*) [24], a secreted triacylglycerol lipase (*gehA*) [25], [26] and two highly immunoreactive cell surface antigens (*htaA*; *hsp20*). The latter includes a protein similar to the product of the *Corynebacterium diptheriae htaA* gene that encodes an iron regulated hemin-binding protein [15] and a heat shock protein (*hsp20*) that encodes an alpha-crystallin-like protein which is homologous to the immunodominant antigen HspX of *Mycobacterium tuberculosis* [26]. For comparison, we also included a putative haemolysin/cytotoxin (*tly*) gene and a family of five co-haemolytic Christie-Atkins-Munch-Peterson (*camp*) factor homologue genes [12], [14] Previous population genetic studies of *P. acnes* demonstrated that phylogenetic trees based on *tly* and CAMP factor gene sequences correctly cluster isolates into the main genetic divisions (I, II and III), suggesting they have co-evolved with housekeeping genes [12], [14]. To date, the precise biological function of CAMP factors and their role in bacterial virulence is unclear, although an ability to function as immunoglobulin-binding proteins or pore-forming toxins have been suggested [27]. In the case of *P. acnes*, the five CAMP factor homologues are likely to have arisen by multiple gene duplication events resulting in paralogous sequences that now encode proteins with unknown and divergent functions (Figure S1). Previously described genes encoding immunogenic dermatan-sulfate binding adhesins (DsA1; DsA2) with putative phase/antigenic variation signatures were not included in the analysis due to the potential for within clone variation in sequences encoding PT repeat regions [15], [17].

**Table 1:** Characteristics of *P. acnes* ‘putative virulence’ genes based on data from whole genome sequencing projects.

Gene	Size (bp)analysed	Genomic location^a^	No. of alleles	No. polymorphic sites	% polymorphic sites	N/S^b^	G+Cmol	*d_N_d_S_*	θ	π	Tajima’s *D* test^c^
*camp 1*	858	1462006–1462863	9	42	4.89	20/22	58.83	0.310	0.018	0.018	0.052
*camp 2*	804	756689–757492	13	37	4.60	20/17	59.40	0.263	0.015	0.017	0.810
*camp 3*	816	2282705–2283520	8	16	1.96	6/10	63.83	0.221	0.008	0.006	−0.975
*camp 4*	802	1339073–1339876	10	28	3.48	9/19	58.10	0.091	0.012	0.014	0.576
*camp5*	846	1305348–1306193	11	27	3.19	11/16	58.15	0.178	0.011	0.011	0.264
*Tly*	777	1514498–1515273	11	32	4.12	16/16	58.31	0.346	0.014	0.012	−0.517
*gehA*	1020	2278518–2279537	9	14	1.37	10/5	63.46	1.180	0.005	0.005	−0.599
*pap60*	1158	793035–794192	16	52	4.49	26/24	60.30	0.219	0.014	0.016	0.692
*pAmce*	933	1057639–1058571	13	41	4.39	13/28	62.29	0.142	0.014	0.013	−0.460
*htaA*	1404	865732–866931	12	34	2.42	15/19	57.86	0.245	0.008	0.008	−0.074
*hsp20*	458	808957–809415	6	12	2.61	3/9	61.75	0.087	0.011	0.013	1.016

a Relates to genome sequence of KPA171202 [28].

b Ratio of non-synonymous-to-synonymous mutations (ω).

c p>0.10 for all Tajima’s *D* test results.

The number of distinct alleles, polymorphic sites and ratio of non-synonymous-to-synonymous mutations (*d_N_d_S_*) were initially investigated for each candidate gene using data currently available from the Human Microbiome Project (HMP) [Huiying Li (2010); http://precedings.nature.com/documents/5305/version/1] as well as completed genome sequencing projects for the isolates KPA171202 (type IB) [28], 6609 (type IB) [29] and ATCC11828 (type II) [30]. HMP isolates represented by HL037PA2, HL037PA3 and HL044PA1 were not included in our analyses as these organisms have recently been proposed as a new species, *Propionibacterium humerusii* [31]. We also observed that the isolate SK182B-JCVI shared only 91% identity to *P. acnes* based on *recA*, whereas the main genetic divisions within *P. acnes* (I, II and III) are 98–99% identical based on this locus, suggesting that it may also represent a novel species despite the high 16 S rRNA sequence identity (99%). On this basis, it was also excluded.

With this cohort of isolates, the number of individual alleles for the genes varied from six (*hsp20*) to 16 (*pap60*), with the number of polymorphic sites ranging from 12 (2.61%; *hsp20*) to 52 (4.49%; *pap60*). Overall, the level of diversity was low as reflected in the θ and π values calculated for each gene. The proportion of polymorphic sites was similar to that previously found with core housekeeping genes [17]. With the exception of the *gehA* gene, all candidate loci had *d_N_d_S_* <1 indicating stabilising selection, although with *camp2* and *pap60* genes a slight increase in the number of non-synonymous versus synonymous changes was observed. Collectively, the results obtained are consistent with previous studies of *tly* and CAMP factor gene sequences which had polymorphic changes consistent with a lack of selection for enhanced diversification [12], [14], [21]. While the *d_N_d_S_* ratio>1 observed with *gehA* is suggestive of a possible role for diversifying or positive selection in the history of this gene, the Tamija’s *D* value for *gehA* and all other loci did not deviate significantly from zero (p>0.10) which was consistent with neutral (random) evolution (Table 1). Tajima’s *D* test is based on the differences between the number of segregating sites (θ) and the pairwise nucleotide diversity (π). Furthermore, analysis of individual codons for positive selection using the single likelihood ancestor counting (SLAC) method with the General Reversible (REV) and HKY85 models of nucleotide substitution did not provide any evidence for positively selected sites within the lipase gene.

### Selection of Virulence Genes for eMLST

From our initial analyses, four loci were selected and investigated for their suitability in an eMLST scheme; these were genes encoding *tly*, CAMP factor homologue 2 (*camp2*), *gehA* and *pap60*. Complete rather than partial gene sequences were examined to maximise discriminatory power. For comparative purposes, all 71 isolates from the HMP and other whole genome sequencing projects were initially analysed using our previously described MLST scheme which is based on partial sequences from seven core housekeeping genes [17]. A total of 21 STs were identified using the originally described method and included 14 STs from the type I division (RAPD group 01) (type IA & IB), which split into nine STs representing the type IA_1_ cluster, three STs representing type IA_2_, one ST representing the type IB grouping, and a novel ST placed within the broader type I clade pending further analysis. A total of seven STs were from the type II division (RAPD group 02). Interestingly, no type III isolates (RAPD group 03) were found in the HMP *P. acnes* collection. Type IA_1_ STs were represented by ST1 (n = 1), ST6 (n = 32), ST11 (n = 1), ST25 (n = 1), ST39 (n = 1), ST49 (n = 2), ST50 (n = 1), ST57 (n = 1) and ST58 (n = 1); type IA_2_ STs by ST9 (n = 15), ST22 (n = 1), ST45 (n = 1); all type IB isolates by ST10 (n = 3) and the novel genotype by ST29 (n = 1). Type II STs were represented by ST35 (n = 2), ST40 (n = 1), ST42 (n = 2), ST44 (n = 1), ST46 (n = 1), ST47 (n = 1) and ST48 (n = 1).

All of the *P. acnes* isolates were then analysed by eMLST with the addition of the complete gene sequences from *tly* (777 bp), *camp2* (858 bp) and *gehA* (1020 bp), to give an initial 10 locus-based scheme (5736 bp). The *pap60* gene was not included in this preliminary analysis due to its adjacent genomic location to the *camp2* locus. In total, 30 eMLST STs (eSTs) were resolved. Isolates represented by ST6 were sub-divided into nine distinct STs. The replacement of *camp2* with *pap60* did not provide any significant improvement in subtyping and consequently, the *camp2* locus was retained due to its smaller size and therefore suitability for nucleotide sequence analysis (data not shown). When all the isolates were then analysed with only the *tly* and *camp2* genes present (lipase gene removed) the same number of eSTs (n = 30) were obtained. Furthermore, although the *recA* gene has proved a robust and valuable locus for broad discrimination of the major clades or phylogroups (I, II and III), its removal did not result in any reduction in the number of STs resolved, or change the clustering observed upon eBURST analysis (data not shown). On this basis, an MLST methodology based on *aroE, atpD, gmk, guaA, lepA, sodA*, *tly* and *camp2* sequences was adopted. The *P. acnes* MLST database has now been updated to include all novel allele sequences for *tly* and *camp2* putative virulence determinants, along with new allelic profiles and ST designations based on the eight-locus scheme (http://pubmlst.org/pacnes).

### Comparison of Original MLST and eMLST Schemes for Resolution of STs

To date, the original *P. acnes* MLST database comprises 62 STs from the analysis of 314 isolates, including all HMP isolates. These isolates have been recovered from acne, medical device, ophthalmic, dental and soft tissue infections, and healthy skin, and cover all four continents. For a more comprehensive comparison of our original and expanded MLST schemes, a total of 285 isolates (including all HMP and other whole genome sequence strains) from our current isolate database were analysed (Table S1). These isolates were selected to represent all 62 STs recovered from a wide range of sources and geographical regions, and included 145 isolates from the type IA_1_ division, 32 isolates from type IA_2_, four novel type I isolates, 40 type IB isolates, 38 type II isolates and 26 type III isolates.

With the eMLST method, a total of 91 eSTs were identified demonstrating additional resolution (Table S1). A total of 42 eSTs were resolved from the type IA_1_ division, seven eSTs from type IA_2_, nine eSTs from type IB, two eSTs from the novel type I group, 22 eSTs from type II and nine eSTs from type III. In particular, isolates represented by ST6 (n = 105) were subtyped into a further 14 additional eSTs, with eST1 representing the most predominant genotype (n = 50; 48%) followed by eST3 (n = 26; 25%), eST4 (n = 15; 14%) and eST8 (n = 4; 4%). The remaining 10 eSTs only represented one isolate each. In the development of our previous method we found little additional resolution when we examined a subset of ST6 strains with nine partial gene sequences versus seven; and in this study we found that a large proportion of the ST6 isolates we previously described retained the same ST (eST1) upon eMLST analysis [17].

With the widespread type IB genotype ST10 identified using our original scheme we found much less variation. Of 36 ST10 isolates examined, 32 retained the same ST with the expanded scheme (eST5). Similarly, with 22 ST9 isolates from the type IA_2_ cluster, 20 retained the same ST (eST2). As the type IB and IA_2_ divisions represent tight monophyletic clusters, there is significantly less opportunity for enhanced discrimination and this is supported by phylogenomic analysis of multi-housekeeping gene datasets compiled from completed and currently ongoing *P. acnes* whole genome sequencing projects [18]. With respect to the large type I clade, a total of 60 eSTs were identified versus 38 STs using the original seven locus scheme [17].

### eBURST Clustering of STs

**Figure 1:**
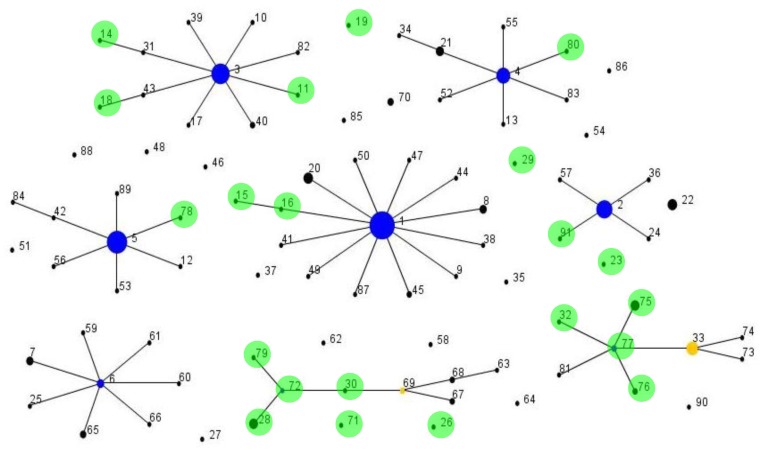
eBURST population snapshot of the eMLST database. A total of eight clonal complexes, where isolates share 7/8 loci with at least one other eST in the group, and 21 singletons were identified. The frequency of each eST is indicated by the area of each circle. Type IA_1_ isolates are represented by CC1, CC3, CC4 and 11 singletons; type IA_2_ by CC2 and two singletons, type IB by CC5 and one singleton; type II by CC6, CC69 and six singletons; type III by CC77 and one singleton. Founding genotypes are highlighted in blue and sub-founders in yellow. eSTs only isolated from healthy skin are circled in green. Note, the spacing between singletons and clonal complexes is not related to the genetic distance between them. Culture collection strain NCTC737 (type IA_1_) is represented by eST1 (ST18, Arahus), KPA171202 by eST5 (ST36, Arahus), CCUG32901 by eST5 (ST36, Arahus) and ATCC11828 by eST27 (novel ST, Arahus).

We also compared the original and expanded schemes with respect to the clustering of isolates using the eBURST algorithm. The 62 STs identified using our original scheme clustered into a total of seven eBURST groups or clonal complexes (CCs), along with two singletons, under the strict definition of sharing 6/7 alleles with at least one other ST (data not shown). With the expanded eight locus scheme, 91 eSTs clustered into a total of eight CCs and 21 singletons based on sharing 7/8 alleles with at least one other ST (Figure 1). All isolates that would be classified as ST6 based on the original seven locus scheme were split into a further three CCs (CC1, CC3, CC4) by eMLST. All 15 STs previously described within the large and dominant type IA clonal complex CC6 [17] either split into these three CCs or were singletons (n = 3).

Isolates that cluster within the type IA_2_ division formed their own CC (CC2) along with three singletons (ST22; ST23) confirming their distinct nature. In our previous MLST study, STs that cluster within this group were provisionally placed in the IB division (IB_2_) pending more detailed analysis [17]. This classification was based on collective criteria including, a type IB biotype, eBURST clustering with known type IB strains, and the presence of various housekeeping and virulence gene alleles matching those found in all type IB isolates; some of the latter loci being used to identify strains from the type IB division by single locus phylotyping [6], [12], [32]–[34]. Furthermore, in our previous study of 54 acne isolates, no type IA_2_ strains were identified, unlike other type IA STs, suggesting differences in infection profile [17]. Recent whole genome sequencing of three type IA_2_ isolates (eST2, eST22 and eST36) has confirmed their classification within the type IA division, but highlighted consistent genetic differences from other type IA strains leading to their proposal as type IA_2_, with all other type IA isolates as IA_1_ [19]. Their classification as a highly distinct division within type IA is also supported by phylogenomic analysis of multi-housekeeping gene datasets compiled from completed and currently ongoing *P. acnes* whole genome sequencing projects [18]. Strains from the type IB division have also been shown to contain 86 genes not present in type IA_2_ organisms, including those involved in carbohydrate metabolism and genes encoding a bacteriophage [18]. The observation of type IB housekeeping and ‘putative virulence’ gene sequences in the type IA_2_ group, which span a distance of at least ∼420 Kbp, therefore suggests conjugal transfer of very large genomic fragments in the history of this cluster [17], [21].

### Selective Pressure

To date, a total of 23 novel *tly* and 33 novel *camp2* alleles have been identified from 285 isolates, with the *camp2* gene currently representing the greatest number of alleles within our MLST database. We also identified a novel *camp2* allele (allele 33) with an additional three bp insertion (GGG), likely reflecting slipped strand mispairing, which did not affect the reading frame of the gene. With our additional sequence data, the number of percentage polymorphic sites for the *tly* and *camp* genes increased to 6.3% and 9.7%, respectively. The updated *d_N_d_S_* ratio for both genes was still consistent with purifying selection (*tly*; 0.299) (*camp2*; 0.295) and Tajima’s *D* values with neutral (random) evolution (*tly*; −0.337, p>0.01) (*camp2*; 0.150, p>0.01).

To corroborate these results and explicitly demonstrate that the *tly* and *camp2* genes are not under positive or diversifying selection, we conducted a number of further analyses. We firstly examined our data using the tests of selection described by Fu and Li (Ref) (D* and F*) which examines the hypothesis that all mutations are selectively neutral and uses the number of singletons (mutations appearing only once) to infer changes at the tips of a phylogeny relative to the total number of changes. The D* test statistic is based on the differences between the number of singletons and the total number of mutations. The F* test statistic is based on the differences between the number of singletons and the average number of nucleotide differences between pairs of sequences. The D* and F* statistics for the *tly* (−0.311, −0.374, respectively) and *camp2* (−0.114, −0.030, respectively) genes were not significant (p>0.1), again consistent with neutral evolution. SLAC analysis of the data with REV and HKY85 models of substitution also provided no evidence for positive selection of any amino acid (codon) in either protein. We then analysed our data using both Mixed Effects Model of Episodic Selection (MEME) and PARtitioning for Robust Inference of Selection (PARRIS) methods. The MEME program is capable of identifying both episodic and pervasive positive selection at individual sites and models *d_N_d_S_* across lineages at an individual site using a two-bin random distribution. PARRIS robustly identifies overall signatures of selection by using a partitioning approach and site-to-site variation in synonomous and non-synonymous rates after accounting for recombination using the GARD algorithm. With both approaches, no evidence of episodic diversifying selection was found in either gene (p>0.1) using both REV and HKY85 models of substitution, thus confirming the previous tests.

It is clear these genes are not under diversifying selection and have co-evolved with housekeeping genes. Their presence in all *P. acnes* isolates provides evidence of their importance for commensal existence, and a selective advantage in the ‘non-pathogenic’, as well as ‘pathogenic’ life of the bacterium preventing their loss from the population. On this basis, we could consider these genes as part of the core genome of *P. acnes*. Various functions have been ascribed to type III haemolysins, and they are also associated with other non-pathogenic propionibacteria such as *Propionibacterium freudenreichii* subsp. *shermanii* [1].

### Recombination

Evidence for recombination was initially sought by construction of ME trees for both loci which should be congruent in a clonal population. The trees generated were essentially concordant with those previously obtained using housekeeping loci with the major divisions (I, II and III) forming distinct and highly significant clades (Figure S2). Some evidence for limited recombination within the type I division was identified; in particular *tly* allele 8 which was present in all type IB and 97% type IA_2_ isolates was also shared with 18% of type IA_1_ isolates. The *camp2* alleles 1 and 6 were shared between different isolates of type IA_1_ and IB. Split decomposition analysis provided further evidence for recombination events due to the presence of interconnected pathways or parallelogram structures, but this was very limited and not statistically significant (phi test; p>0.5) (Figure S3). Furthermore, GARD analysis and Sawyer’s run test found no evidence of significant levels of recombination, similar to previous observations with *P. acnes* housekeeping genes [17], [21].

**Figure 2:**
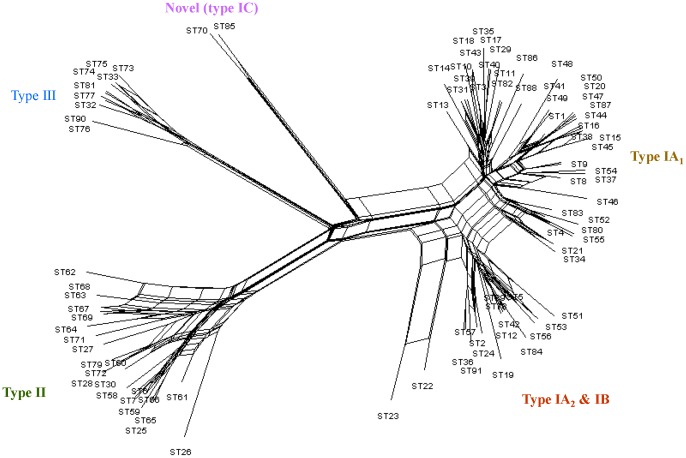
Neighbour-net splits graph of allelic profiles from all 91 *P. acnes* eSTs. Parallelogram structures indicative of recombination are clearly present within the major divisions I, II and III. Overall levels of recombination within the population were statistically significant (phi test; p = 0.021).

Previously, we found that the *P. acnes* population as a whole was clonal and in linkage disequilibrium [17]. In this study, we re-examined our initial analysis using data from the expanded scheme and much larger isolate collection by estimating index of association (*I_A_*) values. An *I_A_* value not significantly greater than zero after 1000 computer randomisations suggests linkage equilibrium, while an *I_A_* value greater than zero is considered clonal. When all isolates were analysed, we obtained an *I_A_* value of 0.483 (p<0.001), and with a representative of each ST an *I_A_* value of 0.379 (p<0.001), confirming the clonal population structure. We also took this opportunity to investigate the level of linkage between alleles within the major *P. acnes* divisions (phylotypes I, II and III). With all isolates from the type I clade (IA_1_, IA_2_, IB), an *I_A_* value of 0.155 (p<0.001) was obtained. While still representing a clonal structure overall, the drop in *I_A_* value indicated increased linkage equilibrium within this population. Similarly, although the numbers of type II and type III isolates analysed were smaller, there was also a consistent drop in *I_A_* value to 0.065 (p<0.001) for the type II population and 0.025 (p = 0.104) for type III. The latter was not statistically significant indicating linkage equilibrium. These results provide evidence to support the idea that the different phylogroups of *P. acnes* may occupy distinct ecological niches, where recombination is more frequent amongst individuals of the same niche, but less frequent between isolates from different niches [35]. Split graph analysis of allelic profiles from all 91 eSTs provided further evidence for recombination events, primarily within the major divisions (multiple interconnected pathways), which were resolved into highly distinct clusters within the network tree structure (Figure 2). Analysis of the population as a whole revealed levels of recombination that were statistically significant (phi test; p = 0.021).

### Phylogenetic Analysis

**Figure 3:**
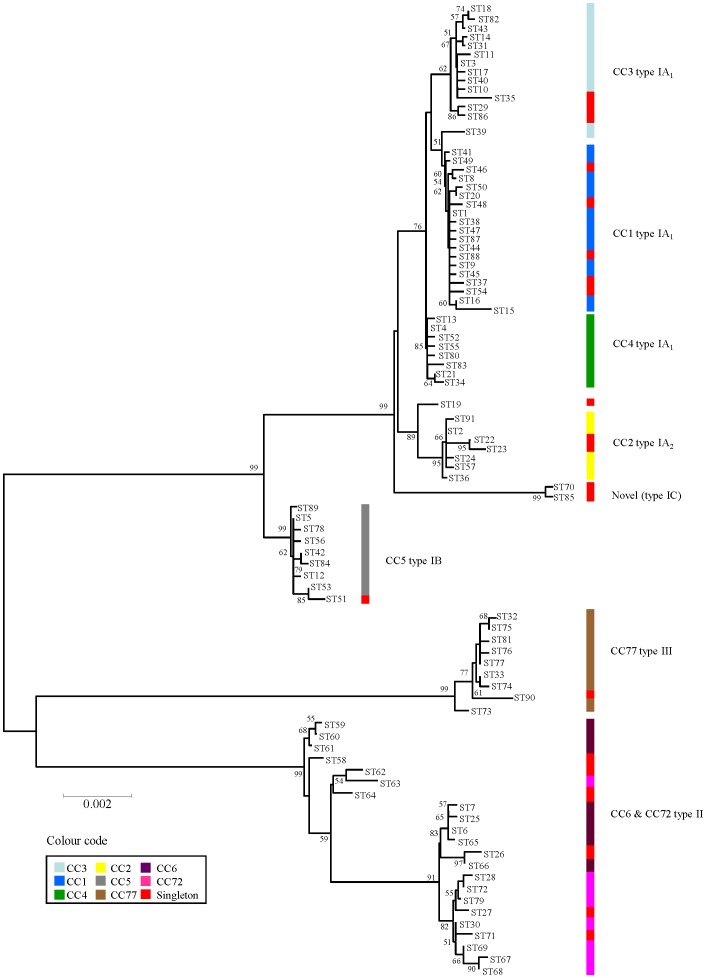
Minimum evolution phylogenetic tree of concatenated gene sequences from all 91 *P. acnes* eSTs. The tree was constructed using concatenated sequences (4253 bp) from each eST. The sequence input order was randomized, and bootstrapping resampling statistics were performed using 500 data sets. Bootstrap values are shown on the arms of the tree. Horizontal bar represents genetic distance. Coloured vertical bars on the right relate to eBURST groupings or clonal complexes. The colour scheme relating to each eBURST group is described, with singletons highlighted in red. Culture collection strain NCTC737 (type IA_1_) is represented by eST1 (ST18, Arahus), KPA171202 by eST5 (ST36, Arahus), CCUG32901 by eST5 (ST36, Arahus) and ATCC11828 by eST27 (novel ST, Arahus).

As the *tly* and *camp2* genes are under purifying selection, do not display statistically significant levels of reticulate evolution, and contain phylogenetically valuable information, they can also be used in combination with the housekeeping genes for investigation of phylogenetic relationships, as previously described with other virulence-related genes [36], [37]. To investigate this, we constructed an ME concatenated gene sequence tree for all 91 eSTs (Figure 3). The ME algorithim was chosen to enable direct comparison with previously described trees in the literature [18], [21]. Consistent with our original MLST scheme, isolates were neatly resolved into three major clades that corresponded to the main genetic divisions I, II and III (bootstrap 99%) [17]. Furthermore, within the type I clade, all isolates representing type IB STs formed a highly distinct cluster (99% bootstrap) that was well separated in genetic distance from type IA strains (Figure 3).

To assess the reliability of our eMLST method for sequence-based clustering of strains within the large type IA clade, we compared the topology of our tree with a recently published *P. acnes* ME reference tree that was constructed using the concatenated sequences of 76 housekeeping genes (92,577 bp) extracted from a total of 75 genomes (mostly from the HMP), the majority of which represent type IA STs (84%) along with a small number of type IB (5%) and type IIs (11%) [18]. As expected, the small number of representative type IB and type II STs in this collection formed highly distinct clades (100% bootstrap values) that matched the topology of our eMLST tree (Figure 3). The clustering of strains within the type IA division was also found to be highly congruent between our eMLST tree and the published reference tree [18]. In particular, type IA isolates clustered into two highly distinct groups (≥75% bootstrap values) that corresponded to the recently proposed type IA_1_ and type IA_2_ divisions [19]. Within our eMLST tree, the majority of type IA_1_ isolates (∼95%) clustered into three groups that corresponded to the clonal complexes CC1 (CC18, Aarhus), CC3 (CC3, Aarhus), CC4 (CC31, Aarhus), and this again matched the topology of the reference tree (Figure 3) [18].

In contrast to all other HMP isolates, the published *P. acnes* reference tree identified that HL097PA1, SK187, HL025PA1 and HL086PA1 represent distinct lineages [18]. The unique nature of HL097PA1 and SK187 isolates was also disclosed within our eMLST tree (Figure 3). In particular, the isolate HL097PA1 formed a unique cluster (eST70; 3 isolates), along with the isolate PV66 (eST85; 1 isolate), which was highly distinct from all other type IA isolates (Figure 3). Identical clustering was observed on the reference tree [18]. These eSTs were also highly distinct based on the Neighbour-net splits tree of allelic profiles (Figure 2) and were singletons upon eBURST analysis (Figure 1). Isolates within this cluster correspond to the novel type I grouping highlighted earlier. On an ME concatenated gene sequence tree constructed from Aarhus MLST STs, the unique nature of HL097PA1 (Aarhus, ST74) was also disclosed, but the isolate clustered within a distinct type IA clade, along with STs from CC18 (ST29), CC28 (ST70) and CC31 (ST31) (Figure S4). This distinct cluster, previously described as I-1b [21], was not observed on the eMLST tree or the *P. acnes* reference tree [18]. In keeping with the unique status of eST70 and eST85 isolates, we found that they reacted with our previously described type IA (QUBPa1) and type II (QUBPa2)-specific MAbs [12], [17]. These antibodies target cell surface dermatan sulfate-binding adhesins (DsA1; DsA2) and a glycolipid-containing antigen on type IA and type II strains, respectively [12], [17]. Furthermore, these isolates contain unique *recA* and *tly* alleles (Table S1). While all isolates identified as type IC to date are resistant to tetracycline and erythromycin due to rRNA mutations (Table 2), it is too early to ascertain if mutational resistance will be a defining feature of further STs within this cluster. Based on their unique phylogenetic and phenotypic properties, as well as potential clinical importance, we now formally propose this group as type IC.

**Table 2:** Summary of eMLST, monoclonal antibody typing and antibiotic susceptibility testing results for antibiotic-resistant *P. acnes* strains.

								rRNA mutation(s)		MIC^b^			
Isolate	Phylotype	Region	Source	Antibodytyping^a^	Allelic profile	eST	CC	16 S	23 S	TC	EM	CL	Erm(X)
PRP-062	IA_1_	Italy	Acne	IA	1-1-1-9-1-1-1-1	49	CC1	−	+ (2058)	32	≥256	32	–
308.2	IA_1_	Sweden	Acne	IA	1-1-1-3-1-1-2-2	3	CC3	+	−	8	0.032	0.025	–
322.2	IA_1_	Sweden	Acne	IA	1-1-1-3-1-1-2-2	3	CC3	+	−	8	≥256	1	–
401.5	IA_1_	Sweden	Acne	IA	1-1-1-3-1-1-1-2-2	3	CC3	+	+ (2057)	≥256	4	0.5	–
411.2	IA_1_	Sweden	Acne	IA	1-1-1-3-1-1-1-1	1	CC1	+	+ (2058)	8	≥256	16	–
413	IA_1_	Sweden	Acne	IA	1-1-1-3-1-1-17-33	88	S	+	−	8	0.032	0.032	–
416.1	IA_1_	Sweden	Acne	IA	1-1-1-3-1-1-2-2	3	CC3	+	+ (2059)	8	≥256	2	–
423.1	IA_1_	Sweden	Acne	IA	1-1-1-3-1-1-2-2	3	CC3	+	−	8	0.032	0.5	–
423.3	IA_1_	Sweden	Acne	IA	1-1-1-3-1-1-2-2	3	CC3	+	−	8	0.032	0.25	–
425.1	IA_1_	Sweden	Acne	IA	1-1-1-3-1-1-2-2	3	CC3	+	+ (2058)	4	≥256	16	–
429.1	IA_1_	Sweden	Acne	IA	1-1-1-3-1-1-2-2	3	CC3	+	-	8	0.5	0.5	–
R11883	IA_1_	UK	Blood	IA	1-1-1-3-1-1-2-2	3	CC3	+	+ (2058)	6	≥256	32	–
R20767	IA_1_	UK	Pus	IA	1-1-1-3-1-1-2-2	3	CC3	+	+ (2058)	6	≥256	8	–
PRP-002	IA_1_	Australia	Acne	IA	1-1-1-3-1-1-2-2	3	CC3	+	+ (2058)	8	≥256	8	–
PRP-003	IA_1_	UK	Acne	IA	8-1-1-3-1-1-2-2	43	CC3	+	−	24	<0.016	0.016	–
PRP-004	IA_1_	UK	Acne	IA	1-1-1-3-1-1-1-1	1	CC1	+	−	8	<0.016	0.023	–
PRP-053	IA_1_	Australia	Acne	IA	1-1-1-3-1-1-2-2	3	CC3	+	+ (2058)	3	≥256	1.5	–
PRP-101	IA_1_	USA	Acne	IA	1-1-1-3-1-1-2-2	3	CC3	+	+ (2059)	≥256	≥256	1.5	–
PRP-102	IB	USA	Acne	Atypical	1-1-1-4-1-4-8-21	42	CC5	+	+ (2059)	12	≥256	0.5	–
PV66	IC	UK	Acne	Atypical	9-1-5-8-6-8-14-6	85	S	+	+ (2058)	≥256	≥256	24	–
PRP-038	IC	UK	Acne	Atypical	9-1-4-8-6-8-14-14	70	S	+	+ (2059)	32	≥256	0.38	–
PRP-039	IC	UK	Acne	Atypical	9-1-4-8-6-8-14-14	70	S	+	+ (2059)	≥256	≥256	0.38	–
PRP-047	II	Greece	Acne	II	1-4-2-4-2-3-10-10	61	CC6	−	+ (2059)	0.25	≥256	2	–
PRP-078	IA_1_	Japan	Acne	IA	1-1-1-3-7-1-22-2	86	S	−	+ (2059)	0.125	≥256	24	–
434	IA_1_	Sweden	Acne	n/d	5-1-1-3-1-1-1-1	20	CC1	−	−	≥256	≥256	nd	–
226	IA_1_	Sweden	Acne	n/d	1-1-1-3-1-1-2-3	31	CC3	−	−	8	≥256	nd	–
HL097PA1	IC	USA	Acne	n/d	9-1-4-8-6-8-14-14	70	S	+	+ (2058)	nd	nd	nd	–
HL045PA1	IA_1_	USA	Acne	n/d	1-10-1-3-1-1-2-2	17	CC3	+	+ (2058)	16	≥256	≥256	–
HL007PA1	IA_1_	USA	Skin	n/d	1-1-1-3-1-1-2-2	3	CC3	+	+ (2058)	24	≥256	≥256	–
HL038PA1	IA_1_	USA	Acne	n/d	1-1-1-3-1-1-2-4	10	CC3	+	+ (2059)	4	≥256	0.875	–
HL099PA1	IA_1_	USA	Acne	n/d	1-1-1-3-1-1-2-2	3	CC3	+	+ (2058)	6	≥256	≥256	–
HL053PA1	IA_1_	USA	Acne	n/d	1-1-1-3-1-1-2-2	3	CC3	+	+ (2058)	6	≥256	≥256	–
HL056PA1	IA_1_	USA	Skin	n/d	1-1-1-3-1-1-2-2	3	CC3	+	+ (2058)	16	≥256	≥256	–
HL074PA1	IA_1_	USA	Skin	n/d	1-1-1-3-1-1-2-2	3	CC3	+	+ (2058)	24	≥256	≥256	–
HL096PA2	IA_1_	USA	Skin	n/d	1-1-1-3-1-1-2-2	3	CC3	+	+ (2058)	6	≥256	≥256	–
HL043PA1	IA_1_	USA	Acne	n/d	1-1-1-3-1-1-2-2	3	CC3	+	+ (2058)	8	≥256	≥256	–
HL043PA2	IA_1_	USA	Acne	n/d	1-1-1-3-1-1-2-2	3	CC3	+	+ (2058)	3	≥256	≥256	–
HL072PA1	IA_1_	USA	Acne	n/d	1-1-1-3-1-1-1-1	1	CC1	+	+ (2058)	12	≥256	≥256	–
HL072PA2	IA_1_	USA	Acne	n/d	1-1-1-3-1-1-1-1	1	CC1	+	+ (2058)	6	≥256	≥256	–
HL005PA1	IA_1_	USA	Skin	n/d	1-1-1-3-1-1-5-2	11	CC3	+	+ (2058)	8	≥256	≥256	–

a Antibody typing with type IA and type II monoclonal antibodies QUBPa1 and QUBPa2, respectively [12].

b Tetracycline resistance MIC≥1.0 mg/L; erythromycin resistance MIC≥0.5 mg/L; clindamycin resistance MIC ≥0.25 mg/L.

With respect to SK187, it had a distinct ST (eST19) that clustered separately from all other type IA isolates, and was also a singleton upon eBURST analysis (Figure 1). The distinct nature of this isolate was also previously reported by our seven gene MLST scheme (ST39) [17]. In the Aarhus scheme, the unique nature of SK187 was not detected as it shared the same ST (Aarhus, ST67) with HL037PA1 and clustered within the Aarhus type IA_2_ clonal complex CC28 (Figure S4) [18]. SK187 has previously been shown to share a genomic region with the type IB strains KPA171202 and HL030PA1, which encodes ABC transporters, conjugal transfer systems and lanthionine biosynthesis [26]. It also shares many gene alleles with type IA_2_ isolates including *recA*. In this study we classified SK187 within the type IA_1_ group based on its phylogenomic clustering [18].

The unique nature of HL025PA1 or HL086PA1 was not disclosed by the eMLST scheme, which classified both isolates as eST4 (CC4) (Figure 2). While the Aarhus MLST scheme did resolve the isolates into unique STs (ST27; ST31) their aberrant genetic nature was similarly not detected and both isolates belonged to CC28 (equivalent to eMLST CC4) (Figure S4) [18].

### Comparison of eMLST and Aarhus MLST Schemes

We compared our eight locus eMLST scheme with the nine locus Aarhus scheme based on a range of criteria. Firstly, using data currently available in the MLST databases, we calculated that the mean number of alleles per locus based on the eMLST scheme is 17.8 compared to 12.1 for the Aarhus method. This provides the opportunity for the resolution of 1×10^10^ and 5×10^9^ genotypes, respectively. We then examined the two schemes for the number of STs resolved and eBURST clustering based on using a panel of 86 isolates, which included 70 from the HMP and other whole genome sequencing projects, and 14 Danish isolates described in the original Aarhus MLST publication [21]. During our analyses we noted a significant number of inconsistencies between our ST designations and those recently reported by Kilian and coworkers using the Aarhus MLST scheme [18] [we also noted other inconsistencies in ST designations within the Aarhus database (http://pacnes.mlst.net/)]. These differences, which are highlighted in Table S2, result in an overrepresentation of unique Aarhus STs in their publication [18]. Overall, the eMLST scheme discriminated a greater number of STs (33 versus 28), and CCs (7 versus 6) compared to the Aarhus method (Table S2). The eMLST scheme also identified a greater number of singletons compared to the Aarhus protocol (7 versus 2) (Table S2). Both methods were essentially concordant with respect to the clustering of the 86 isolates into CCs, although a small number of exceptions were noted (Table S2). In addition to the aberrant isolates described earlier, isolates HL030PA2 and HL063PA2 were identified as singletons based on eMLST, but clustered within CC28 based on the Aarhus method [18]. Their distinct nature from strains within CC28 is supported by the *P. acnes* reference tree and also a distinct 16 S rRNA allele [18]. These isolates were also correctly highlighted as distinct using our previous seven gene MLST scheme [17], [18]. With type II strains, two clonal complexes (CC6; CC72) were identified by eMLST compared to only one clonal complex (CC60) using the Aarhus method.

Data presented in Table S2 also highlights the importance of no longer using *recA* for the sole identification of type IB isolates as previously described, although it still remains a highly robust locus for identification of types I, II and III [6], [12], [32]–[34]. While the method appears 100% sensitive for detection of type IB strains, more in-depth MLST analyses have shown it lacks specificity due to sharing of the type IB allele with type IA_1_ isolates from CC4 and all type IA_2_ isolates (CC2), thus leading to potential misidentification of certain type IA isolates as type IB. All isolates that we previously described as type IB based on *recA* typing have been re-examined by eMLST and their original classification does, however, remain correct [12]–[16].

### Investigation of ‘Pathogenic’ Versus ‘Commensal’ *P. acnes* Strains

Under normal circumstances, *P. acnes* has an important role in maintaining the ecosystem of healthy skin by occupying niches that could otherwise be invaded by pathogenic microorganisms [38]. Despite this positive effect on our health, it is also widely recognised that if given the opportunity, *P. acnes* can cause a range of different infections. What is much less certain is whether this is a characteristic of all *P. acnes* strains, or whether specific pathogenic lineages exist alongside truly benign commensal strains that are only ever associated with maintaining health. To properly address this issue, it is important that we analyse a large cohort of isolates, and not just from acne, but from a wide range of *P. acnes*-associated diseases, as well as healthy skin.

Previously, we found that the MLST genotype ST6 was associated with various infections and conditions, particularly inflammatory acne [17]. We re-visited the association of ST6 with disease by looking at the relationship between STs further resolved from ST6 by subtyping and various infections and conditions (Figure S5). The most prevalent of the genotypes identified, eST1 (n = 50) was associated with moderate-to-severe acne (n = 25; 24% acne-associated isolates) and was globally dispersed. The *P. acnes* type strain NCTC737, isolated from an acne patient in London over 90 years ago, was also eST1 based on our scheme (equivalent to ST18 in the Aarhus scheme). The endocarditis isolate 889, previously used to study the pro-inflammatory reaction of sebocytes and keratinocytes, was also eST1 [16], [39]. In our collection, we also have isolates of eST1 recovered from dental (n = 2) and ophthalmic infections (n = 7) and fatal head granulomas (n = 2) providing further evidence that the pathogeneic profile of this lineage is not just confined to acne (Table S1; Figure S5). Furthermore, eST1 was also isolated from healthy skin (n = 13) demonstrating that it may also be a normal component of the skin microbiota.

The other prevalent MLST genotypes represented by eST3 (n = 26) and eST4 (n = 15) represented 17% and 8% of acne isolates, respectively. These STs were also widely disseminated and found in association with other conditions, including ophthalmic and soft tissue infections as well as healthy skin (Table S1; Figure S5). The remaining STs derived upon ST6 subtyping were isolated from acne, soft tissue infections and skin (Table S1; Figure S5). As expected, no clear differences were observed in the associations between the three clonal complexes (CC1, CC3 and CC4) derived from the previously described CC6 and acne or other clinical conditions; this suggests that although these complexes may indeed differ in genomic content this may not necessarily reflect differences in disease potential [18].

While our previous study found no association between isolates from the type IA_2_ division and acne [17], in this current study with a larger isolate collection we did identify nine type IA_2_ STs recovered from the skin of acne patients. This represents, however, only 9% of all the acne-associated isolates and 28% of the total number of type IA_2_ isolates identified. This rate of recovery from acne patients is similar to that seen with type IB and type II isolates (Table 3). A large proportion of type IA_2_ isolates were associated with healthy skin (n = 14; 44% total) while the remainder were isolated from ophthalmic infections (n = 8; 25% total) and one isolate was associated with an intravenous catheter (n = 1; 3% total) (Table S1; Figure S5). In contrast, within the type IA_1_ division we find that acne isolates account for approximately 50–60% of all the isolates within the three major clonal complexes CC1, CC3 and CC4 (p<0.01). The re-appraisal of IB_2_ isolates as type IA_2_ now means that nearly all cases of acne and ophthalmic infections appear associated with type IA organisms. On this basis, and using the much greater number of clinical isolates now in our collection, we have updated our previously published table [17] highlighting the breakdown of the *P. acnes* phylotypes isolated from acne and ophthalmic infections, and also included data relating to healthy skin (Table 3). It is currently unclear why type IA_2_ isolates appear to be less frequently recovered from acne patients compared with type IA_1_ strains. In particular, all type IA_1_ and IA_2_, but not type IB, II or III isolates, express the dermatan-sulfate binding adhesins DsA1 and DsA2, which have the capacity for phase/antigenic variation [17]. Dermatan sulfate is the predominant glycosaminoglycan in skin and a biological response modifier involved in various processes. While previous studies have suggested that their expression by type IA strains may provide an explanation, at least in part, for their association with acne and the recurrent nature of the disease [17], [26], the infrequent recovery of IA_2_ strains from acne patients despite their capacity to produce these proteins clearly confirms the importance of other factors. In the context of acne, the latter are likely to include lipase, neuraminidase, iron acquisition proteins and inflammation-inducing molecules [15], [16], [21], [25], [26], [39]. Furthermore, the re-appraisal of type IB_2_ isolates as type IA_2_ now confirms that our previously described monoclonal antibody QUBPa1 is type IA-specific, and does not show any reaction with isolates from the type IB division in line with their distinct nature [12], [17].

**Table 3:** Breakdown of phylotypes for normal skin, acne and ophthalmic infections.

			Ophthalmic infections		
Phylotype	Skin	Acne	Keratitis	Endophthalmitis	Eye^a^
IA_1_	30 (39%)	77 (74%)	11 (58%)	4 (40%)	1 (50%)
IA_2_	14 (18%)	9 (9%)	4 (21%)	4 (40%)	0 (0%)
IB	7 (9%)	6 (6%)	2 (10%)	1 (10%)	1 (50%)
IC	0 (0%)	4 (4%)	0 (0%)	0 (0%)	0 (0%)
II	12 (16%)	8 (8%)	1 (5%)	1 (10%)	0 (0%)
III	14 (18%)	0 (0%)	1 (5%)	0 (0%)	0 (0%)
Total	77 (100%)	104 (100%)	19 (100%)	10 (100%)	2 (100%)

a Type of eye infection not available for isolate.

While type IB and type II isolates appear associated with acne and ophthalmic infections in very small numbers relative to type IA_1_, a combined total of 18 out of 23 medical device related infections were associated with isolates from these two divisions (Table S1; Figure S5). Other type IB and II isolates were associated with dental, soft tissue infections and were also isolated from normal skin. Most type III strains were primarily isolated from spinal disc material and normal skin. Collectively, these data are consistent with previous observations suggesting that type IB, II and III strains are more frequently associated with blood, soft tissue and medical implant-related infections [6], [12], [17], [21], [34]. It is unclear, however, if this association is clinically relevant in many cases, and for medical implants, if it indicates a possible tropism for such surfaces. The clinical associations of all the isolates analysed in this study are detailed in Table S1.

In total, 21 STs were identified only on healthy skin and not associated with infection or a clinical sample (Table S1, Figure 1 and S5). Of particular note was CC72 (type II) and CC77 (type III), in which 50% of the isolates appeared health-associated. A proportionately smaller number of STs within CC1, CC3 and CC4 were also associated with healthy skin, but it remains to be determined if these are truly non-pathogenic, while other STs within these complexes have heightened capacity to cause disease. While the exact clinical significance of the isolates from patient’s samples may be unclear in some cases, the identification of apparent healthy skin-associated STs is consistent with a view that pathogenic versus commensal strains of *P. acnes* may indeed exist.

### Population Genetic Analysis of Antibiotic Resistant *P. acnes* Isolates

Acne vulgaris is a disease of the pilosebaceous follicle and has a multifactorial aetiology. The association of *P. acnes* with acne vulgaris has a long history, although the exact role of the bacterium in the pathophysiology of the disease still remains controversial. The successful treatment of acne patients with both oral and topical antibiotics over the last 40 years has provided evidence in support of *P. acnes* involvement in acne, although the direct or indirect anti-inflammatory activity of these agents may also contribute to their effectiveness [2], [38]. As individual treatments can last for months to even years, it is no surprise that strains of cutaneous propionibacteria resistant to the main antibiotics used to treat acne (tetracyclines, erythromycin, clindamycin) have emerged. Amongst the different approaches utilised by *P. acnes* to confer resistance to anti-acne agents, specific point mutations in the rRNA operon represent a major mechanism [40], [41]. *P. acnes* is an ideal candidate for such a strategy as it contains only a small number of copies of the rRNA operon with no evidence of heterogeneity. As a consequence, any point mutations are not compromised by wild-type rRNA and prolonged antibiotic usage positively selects for such mutants. Resistance to tetracyclines is mediated by a single G-to-C base transversion (equivalent base 1058 in *Escherichia coli*) (1058 G>C) in the 16 S rRNA of the small ribosomal subunit [41]. Resistance to erythromycin and clindamycin most commonly occurs via one of three point mutations in genes that encode domain V of 23 S rRNA (peptidyltransferase loop) [40], but in a minority of isolates resistance is associated with the presence of *erm*(X) [42]. A 2058 A>G mutation confers high erythromycin resistance and variable resistance to other macrolides and clindamycin, while a 2057 G>A mutation is associated with low erythromycin resistance. A 2059 A>G mutation confers high resistance to all macrolides and elevated, but variable, resistance to clindamycin. To date, no detailed population genetic study based on MLST has been carried out on isolates containing such rRNA mutations, and how they relate to previously described phylogroups. Within our isolate collection, and including the HMP strains, we identified a total of 38 isolates that have one or more rRNA mutations that would confer antibiotic resistance to anti-acne antibiotics (Table 2). A further two resistant isolates were identified without any rRNA mutations indicating alternate mechanisms for resistance (Table 2). Both isolates were from Sweden and negative for *erm*(X).

Overall, 85% of the resistant isolates belonged to the type IA_1_ group, which was consistent with our data demonstrating that this phylogenetic cluster was highly associated with acne. Only one type IB (PRP-102) and one type II (PRP-047) isolate were identified, recovered from acne patients in the USA and Greece, respectively. PRP-102 demonstrated multiple resistance to all antibiotics tested, while PRP-047 was resistant to erythromycin and clindamycin. No type IA_2_ or type III isolates from our collection were found to carry these mutations, again consistent with their low rate of recovery from acne lesions. All type IC isolates tested to date were resistant to erythromycin and tetracycline as a result of rRNA mutations, with some variability in the nature of their 23 S mutation; two isolates have 2058 A>G and two isolates 2059 A>G mutations in 23 S rRNA (Table 2). At a deeper level of analysis, ∼65% of the resistant isolates belonged to type IA_1_ CC3 and, of these, 84% were eST3 (ST3, Aarhus). Two singletons, 413 (eST88) and PRP-078 (eST86) were also double locus variants of eST3. An intriguing result from this analysis is the relatively low abundance of rRNA mutations in isolates from CC1, which is also strongly associated with moderate-to-severe acne, at least based on culture detection methods. Only six isolates (15%) were identified from this complex, four eST1 (ST18, Aarhus) along with eST49 (PRP-062) and eST20 (434). It should be noted, however, that in acne patients, *P. acnes* within the hair follicle forms large aggregates that may reflect biofilm production [43], which would intrinsically increase antibiotic resistance [44]. It is, however, difficult to draw any definitive conclusions on the clinical significance of these data until a wider prospective study is conducted and a greater number of isolates are analysed. Eight isolates were resistant to tetracycline via the 16 S rRNA 1058 G>C mutation (nearly all Swedish) but were susceptible to macrolides and clindamycin, whereas 26 isolates carried the 1058 G>C mutation in combination with 23 S rRNA 2058 A>G or 2059 A>G conferring resistance to tetracyclines, macrolides and sometimes lincosamides. Notably, isolates from the USA carrying a 2058 A>G mutation were more likely to be highly resistant (MIC ≥256 mg/L) to clindamycin than isolates from elsewhere (MIC 1.5–32 mg/L); this suggests the presence of one or more additional uncharacterised mechanisms conferring resistance to clindamycin in these isolates. *Erm*(X) was not detected, but we cannot rule out the presence of a different *erm* gene. We recognise that our collection does not fully reflect the relative prevalence of different resistant phenotypes and genotypes as described in the literature [45], [46], [47], [48] and that tetracycline resistant and multiply resistant strains are over-represented.

The presence of rRNA mutations in distinct lineages representing types IA_1_, IB, IC and II clearly demonstrates independent antibiotic selection events. This is further supported by the observation of identical rRNA mutations conferring resistance to erythromycin and tetracycline in the closely related cutaneous species *Propionibacterium granulosum* and *Propionibacterium. avidum* [40], [41]. The identification, however, of geographically widespread isolates of eST3, all with the same rRNA mutations (16 S rRNA 1058 G>C; 23 S rRNA 2058 A>G) does highlight the possibility of dominant resistant clones circulating across continents. Further evidence to support the view that these clones may be widely transmitted through populations comes from the isolation of resistant eST3 from healthy adult skin (Table 2). None of the healthy adults from which these strains were isolated were being treated with antibiotics. Variations in MIC values within this particular clonal lineage may also indicate complemetary antibiotic selection events leading to increased resistance in some cases.

An additional number of geographically distinct strains with unique STs were also identified with rRNA mutations. These include the type IA_1_ strain PRP-062 from Italy (CC1, eST49) which had a 23 S rRNA 2058 A>G mutation only, but also demonstrated high tetracycline resistance, and PRP-047 (type II; CC6, ST61) and PRP-078 (type IA_1_; singleton, ST86) from Greece and Japan, respectively which had 23 S rRNA 2059 A>G mutations. One Swedish isolate 401.5 (type IA_1_; CC3, eST3) from a patient in Stockholm had 16 S rRNA 1058 G>C and 23 S rRNA 2057 G>A mutations, the latter being uncommon. Consistent with these sequences, the isolate displayed high tetracycline resistance and lower levels of erythromycin resistance (Table 2). Finally, resistant isolates from Sweden, 434 (type IA_1_; ST20, CC1) and 226 (type IA_1_; ST31, CC3) with wild type rRNA sequences were identified. These strains had high level resistance that was clearly mediated via other mechanisms, although these were not identified as part of this study. Both strains were, however, negative for the presence of the *erm*(X) resistance gene.

### Conclusions

The expanded eight gene MLST scheme described offers high resolution typing of *P. acnes*. When compared against a large panel of test isolates, the method produced an overall level of discrimination that was higher than the nine-gene method developed at the University of Bath, UK and adopted by the Aarhus group [21]. Furthermore, the clustering of isolates based on analysis of both concatenated sequence data and allelic profiles was highly concordant with phylogenies based on phylogenomic analysis, and also correlated with gene contents and putative pathogenic potential.

Application of the high resolution scheme to a large collection of isolates from disparate geographically widespread clinical sources, as well as healthy skin, revealed particular lineages that appeared to have a heightened capacity to cause infection (particularly, eST1, eST3) when compared to other strains that were only ever isolated from healthy skin, particularly certain STs from CC72 (type II) and CC77 (type III). Whether the latter strains truly represent ‘commensal’ lineages still remains to be firmly established, but the ability to stratify *P. acnes* into isolates that are opportunistic pathogens versus those associated with health would have a number of important ramifications. In particular, from a clinical point of view it opens the possibility of developing novel antimicrobial strategies for acne and other *P. acnes*-related diseases if specific mechanisms leading to enhanced virulence potential can be pinpointed. From a diagnostic perspective, the ability to identify pathogenic versus benign strains may aid diagnostic bacteriologists in their attempts to determine whether *P. acnes* recovered from a patient’s sample is clinically meaningful, or simply reflects contamination with commensal microbiota. Furthermore, in the future, the development of molecular tests for high throughput differentiation of *P. acnes* into the different phylotype groupings (IA_1_, IA_2_, IB, IC and III) would be very valuable, and enable stratification of isolates for downstream MLST analysis.

Due to its relatively low number of rRNA operons, mutations in rRNA have been a significant mechanism utilised by *P. acnes* to evolve resistance to anti-acne antibiotics. One key finding from our analysis of such isolates is the apparent dominance of these types of mutation in CC3, particularly eST3 the founder of this complex. In contrast, isolates from CC1 were represented in low numbers despite the high association of this complex with acne, and its founding genotype eST1 being widely disseminated and the most prevalent ST from moderate-to-severe cases of the disease. Our data also provide tentative evidence that multiple antibiotic resistant eST3 is circulating across continents. A prospective MLST study based on a greater cohort of such isolates will hopefully confirm and expand these results.

Our original work on the phylogenetic structure of *P. acnes* identified the main phylotypes or phylogroups termed IA, IB, II and III [12]–[14]. This nomenclature, which has now been refined based on MLST analyses and whole genome sequencing data to include IA_1_, IA_2_, IB, IC, II and III [12]–[14], [17], [19], is supported at the clinical and phenotypic level and has been widely used in the literature. It remains a robust and highly valuable system for classification of *P. acnes* strains. We recommended that in future communications STs and CCs are described in the context of these divisions as this will provide continuity with a large number of previously published studies and avoid unnecessary confusion that will benefit no one.

## Materials and Methods

### Bacterial Strains

A total of 285 *P. acnes* isolates were examined in this study, including the type strain NCTC737 (type IA_1_; facial skin acne isolate) which was obtained from the National Collection of Type Cultures (NCTC), and reference strains ATCC11828 (type II; human abscess) from the America Type Culture Collection (ATCC), CCUG32901 (type IB; human blood) from the Culture Collection, University of Göteborg (CCUG) and KPA171202 (type IB; DSM 16379; contaminated anaerobic culture) which was from the German Collection of Microorganisms and Cell Cultures. Table S1 summarizes all the isolates analyzed in this study (n = 285) and includes those currently comprising the HMP and other published genome sequencing projects [28], [29], [30]. A total of 104 isolates were from patients with acne, 31 were from ophthalmic-related infections (bacterial keratitis and endophthalmitis), 27 were associated with soft-tissue infections, surgical skin wounds and blood, six with dental infections, 13 with spinal disc material, 14 from sonicate prepared from failed prosthetic hip joints and 10 from intravascular catheters. A total of 77 isolates were from healthy skin. The remaining isolates were from a bone infection, contaminated culture and cadaveric skin.

### Bacterial Culture

All bacterial strains were maintained at −80°C in Brain Heart Infusion (BHI) broth, containing 12% (v/v) glycerol, pending analysis. Organisms were cultured on anaerobic blood agar at 37°C in an anaerobic cabinet (Mark 3; Don Whitley Scientific) under an atmosphere of 10% H_2_, 10% CO_2_, 80% N_2_ prior to analysis.

### MLST Analysis

Partial sequences of the housekeeping gene loci *aroE* (424 bp), *atpD* (453 bp), *gmk* (400 bp), *guaA* (493 bp), *lepA* (452 bp), *recA* (463 bp) and *sodA* (450 bp) were amplified using primer pairs and amplification conditions previously described [17]. Complete gene sequences from the ‘putative virulence’ determinants *tly* (777 bp) and *camp2* (804 bp) were amplified using the methods described in McDowell et al. [12] and Valanne et al. [14], respectively. PCR products were purified on MiniElute UF plates (Qiagen) and sequenced as detailed previously [17]. Novel alleles for each locus were assigned a new allele number and distinct allelic profiles assigned a new ST number. All allele sequences are available at (http://pubmlst.org/pacnes).

### Identification of Antibiotic Resistance Genes

Mutations in both 16 S and 23 S rRNA loci conferring antibiotic resistance were identified by PCR using published primers and amplification conditions [46], followed by nucleotide sequencing as previously described [17]. Detection of *erm* genes (*e*rythromycin *r*ibosome *m*ethylase) was investigated using a PCR assay that targets a 167 bp fragment of the gene [46].

### Data Analysis

For ‘putative virulence’ genes, G+C content, the number of polymorphic sites, the average number of synonymous and non-synonymous sites, the average synonymous/non-synonymous site ratio (*d_N_d_S_*), the nucleotide diversity per site (π) and the average number of nucleotide differences per site (θ) were determined using DnaSp version 5 [49]. Phylogenetic relationships were investigated with the Minimum Evolution (ME) algorithm using MEGA v 4.0. Split decomposition trees were generated using SplitsTree version 4.1 [50]. Evidence for recombination breakpoints was assessed using the GARD method [51]. Individual codons were also analyzed for positive selection using the SLAC, MEME and PARRIS methods with the REV and HKY85 models of nucleotide substitution [51], [52], [53]. Index of association values (*I_A_*) were determined following the method of Haubold & Hudson [54] with LIAN v3.5 software. Clonal groups were identified using the eBURST v3 clustering algorithm which will also identify the most likely (i.e., parsimonious) ancestral ST within each clonal complex (http://www.mlst.net) [55].

### Antibody Typing

Monoclonal antibody (MAb) typing by immunofluorescence microscopy (IFM) was carried out as described previously [12]. Isolates were examined for their reactivity with mouse monoclonal antibodies QUBPa1 and QUBPa2, which target strains within types IA_1_ and II, respectively [12], [13], [17].

### Susceptibility Testing

Susceptibility to the antibiotics tetracycline, erythromycin and clindamycin was determined using E-test strips (AB BIODISK, Sweden) according to the manufacturer’s instructions. The MIC values were determined after 48 hours anaerobic incubation at 37°C.

## Supporting Information

Figure S1 **Minimum evolution phylogenetic tree of amino acid sequences from the CAMP factor homologues of the** ***P. acnes*** **type strain NCTC737 (type IA_1_; eST1).** Sequences were analysed using the Dayhoff Point Accepted Mutation (PAM) matrix algorithm and bootstrapping resampling statistics were performed using 500 data sets. The CAMP factor sequences from *Streptococcus pyogenes* (accession no. NP_802366.1), *Streptococcus uberis* (accession no. AAA78910.1) and *Streptococcus agalactiae* (GenBank accession no. NP_736433.1), were used as outgroups.
(TIFF)

Figure S2 **Minimum evolution phylogenetic trees for** *tly* **(A) and** *camp2* **(B) genes.** Both trees were essentially concordant with that previously obtained using housekeeping loci, with the major divisions (I, II and III) forming distinct and highly significant clades (100% bootstrap values). Some evidence for limited recombination within the type I division was identified; in particular *tly* allele 8 which was present in all type IB and 97% IA_2_ isolates was also shared amongst 18% type IA_1_ isolates, while *camp2* alleles 1 and 6 were shared between different isolates of type IA_1_ and IB.
(TIFF)

Figure S3 **Split decomposition analysis of** *tly* **(A) and** *camp2* **(B) allele sequences.** Some evidence of recombination events was apparent due to the presence of multiple pathways that formed parallelogram structures, but this was very limited. There was no statistically significant evidence of recombination using the phi test (*tly*, p = 0.91; *camp2*, p = 0.78).
(TIFF)

Figure S4 **Minimum evolution phylogenetic tree of concatenated gene sequences from 77** *P. acnes* **STs currently comprising the Aarhus MLST database.** Sequence input order was randomized, and bootstrapping resampling statistics were performed using 500 data sets. Bootstrap values are shown on the arms of the tree. Horizontal bar represents genetic distance. Coloured vertical bars on the right relate to eBURST groupings or clonal complexes. The colour scheme relating to each eBURST group is described, with singletons highlighted in red.
(TIFF)

Figure S5 **Qualitative analysis grids highlighting the relationship between** *P. acnes* **eSTs, different clinical sources and healthy skin (highlighted in colours).** (A) eSTs derived from ST6 by subtyping. Soft tissue relates to fatal head granulomas, endocarditis, blood cultures, lagophtalmus, a cancerous prostate and abscess (B) all 91 eSTs derived for 285 isolates. Soft tissue relates to fatal head granulomas, endocarditis, blood cultures, lagophtalmus, lymph nodes, cancerous prostates, abcesses, a pleuropulmonary infection and kidney infection.
(DOC)

Table S1 **eMLST results for all 285** ***P. acnes*** **isolates analysed in this study.** A total of 91 eSTs were generated based on the analysis of eight gene loci.
(DOC)

Table S2 **Comparison of** *P. acnes* **MLST schemes.** The eMLST (eight loci) and Aarhus MLST (nine loci) schemes were compared against a panel of 86 isolates representing different phylogenetic groups of *P. acnes* (IA_1_, IA_2_, IB, IC, II). STs were clustered within the same CC if they shared 7/8 alleles (eMLST) or 8/9 alleles (Aarhus) with at least one other ST.
(DOC)
