# Supplementary figures and images for: An Expanded Multilocus Sequence Typing Scheme for Propionibacterium acnes: Investigation of ‘Pathogenic’, ‘Commensal’ and Antibiotic Resistant Strains
Source: PLoS One. 2012 Jul 30;7(7):e41480. doi: 10.1371/journal.pone.0041480 (PMC3408437; doi:10.1371/journal.pone.0041480)

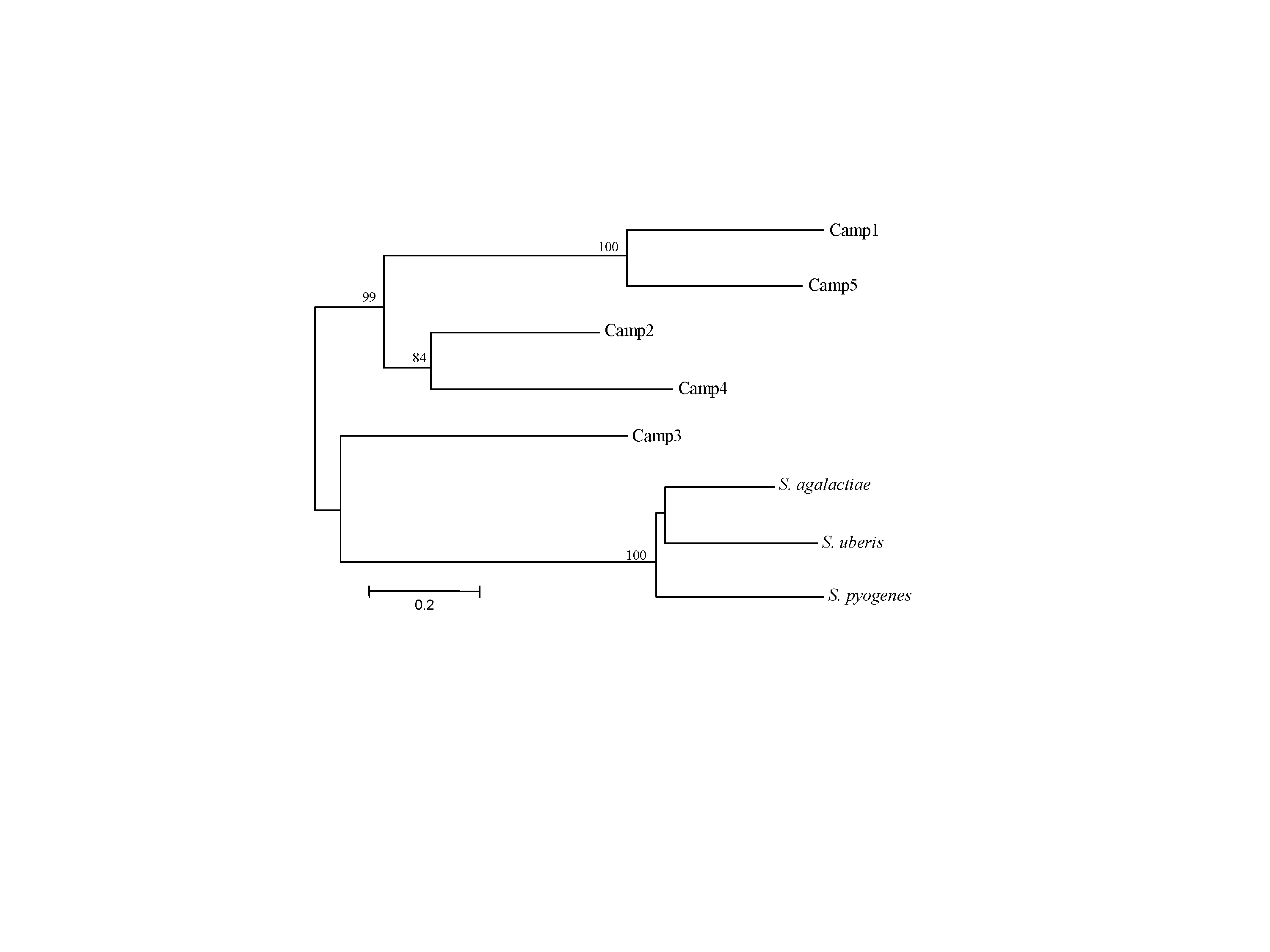

Supplement: Figure S1 — Minimum evolution phylogenetic tree of amino acid sequences from the CAMP factor homologues of the P. acnes type strain NCTC737 (type IA1; eST1). Sequences were analysed using the Dayhoff Point Accepted Mutation (PAM) matrix algorithm and bootstrapping resampling statistics were performed using 500 data sets. The CAMP factor sequences from Streptococcus pyogenes (accession no. NP_802366.1), Streptococcus uberis (accession no. AAA78910.1) and Streptococcus agalactiae (GenBank accession no. NP_736433.1), were used as outgroups. (TIFF) [file pone.0041480.s001.tiff]

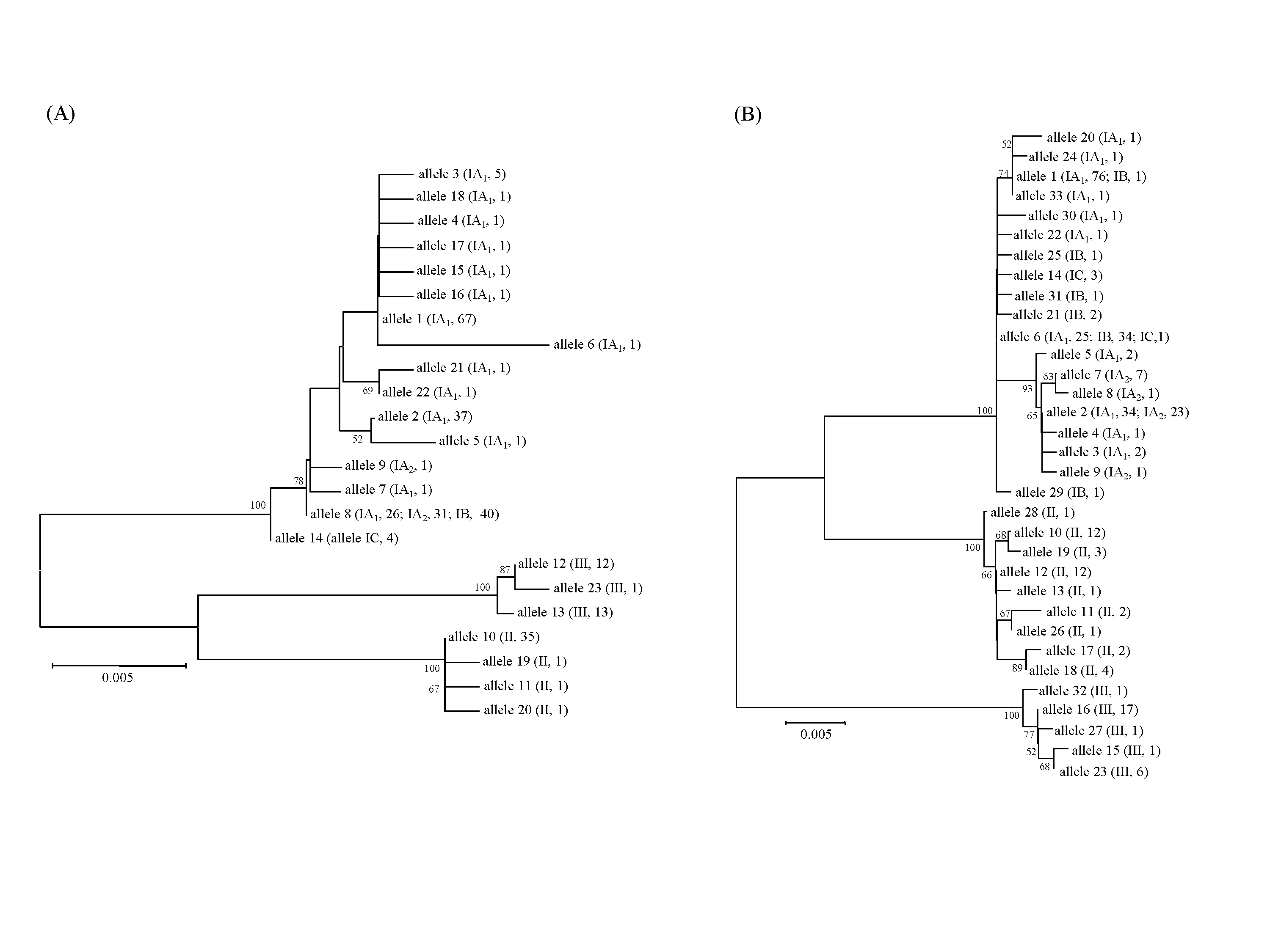

Supplement: Figure S2 — Minimum evolution phylogenetic trees for tly (A) and camp2 (B) genes. Both trees were essentially concordant with that previously obtained using housekeeping loci, with the major divisions (I, II and III) forming distinct and highly significant clades (100% bootstrap values). Some evidence for limited recombination within the type I division was identified; in particular tly allele 8 which was present in all type IB and 97% IA2 isolates was also shared amongst 18% type IA1 isolates, while camp2 alleles 1 and 6 were shared between different isolates of type IA1 and IB. (TIFF) [file pone.0041480.s002.tiff]

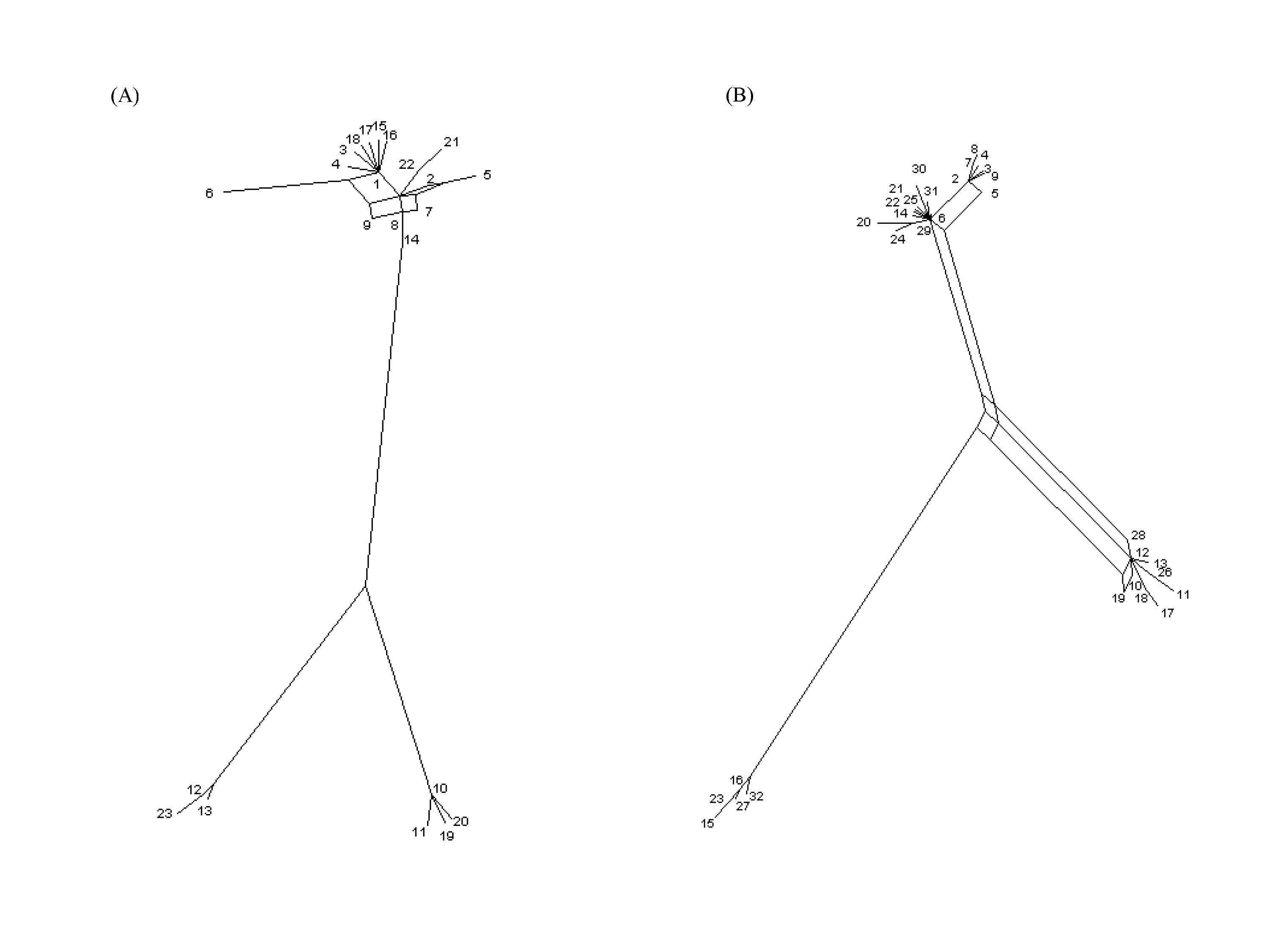

Supplement: Figure S3 — Split decomposition analysis of tly (A) and camp2 (B) allele sequences. Some evidence of recombination events was apparent due to the presence of multiple pathways that formed parallelogram structures, but this was very limited. There was no statistically significant evidence of recombination using the phi test (tly, p = 0.91; camp2, p = 0.78). (TIFF) [file pone.0041480.s003.tiff]

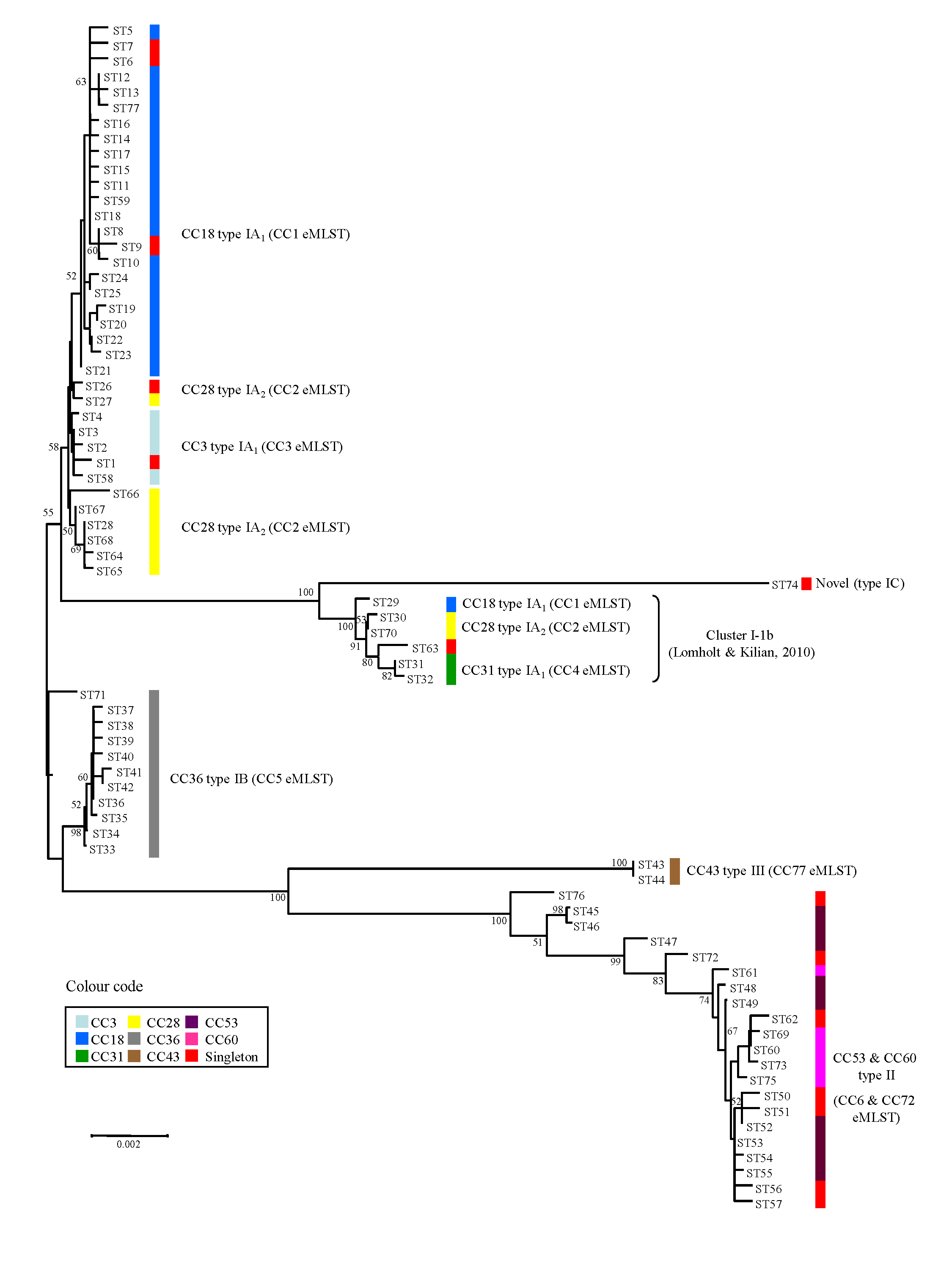

Supplement: Figure S4 — Minimum evolution phylogenetic tree of concatenated gene sequences from 77 P. acnes STs currently comprising the Aarhus MLST database. Sequence input order was randomized, and bootstrapping resampling statistics were performed using 500 data sets. Bootstrap values are shown on the arms of the tree. Horizontal bar represents genetic distance. Coloured vertical bars on the right relate to eBURST groupings or clonal complexes. The colour scheme relating to each eBURST group is described, with singletons highlighted in red. (TIFF) [file pone.0041480.s004.tiff]
