# Supplementary material for: An Expanded Multilocus Sequence Typing Scheme for Propionibacterium acnes: Investigation of ‘Pathogenic’, ‘Commensal’ and Antibiotic Resistant Strains
Source: PLoS One. 2012 Jul 30;7(7):e41480. doi: 10.1371/journal.pone.0041480 (PMC3408437; doi:10.1371/journal.pone.0041480)
Supplement: Figure S5 — Qualitative analysis grids highlighting the relationship between P. acnes eSTs, different clinical sources and healthy skin (highlighted in colours). (A) eSTs derived from ST6 by subtyping. Soft tissue relates to fatal head granulomas, endocarditis, blood cultures, lagophtalmus, a cancerous prostate and abscess (B) all 91 eSTs derived for 285 isolates. Soft tissue relates to fatal head granulomas, endocarditis, blood cultures, lagophtalmus, lymph nodes, cancerous prostates, abcesses, a pleuropulmonary infection and kidney infection. (DOC) [file pone.0041480.s005.doc]

**(A)**

| **ST** | **Acne** | **Ophthalmic** | **Medical device** | **Soft tissue** | **Dental** | **Spinal disc** | **Bone** | **Skin wound** | **Skin** |
| --- | --- | --- | --- | --- | --- | --- | --- | --- | --- |
| **1** |  |  |  |  |  |  |  |  |  |
| **3** |  |  |  |  |  |  |  |  |  |
| **4** |  |  |  |  |  |  |  |  |  |
| **8** |  |  |  |  |  |  |  |  |  |
| **9** |  |  |  |  |  |  |  |  |  |
| **10** |  |  |  |  |  |  |  |  |  |
| **11** |  |  |  |  |  |  |  |  |  |
| **13** |  |  |  |  |  |  |  |  |  |
| **31** |  |  |  |  |  |  |  |  |  |
| **38** |  |  |  |  |  |  |  |  |  |
| **39** |  |  |  |  |  |  |  |  |  |
| **82** |  |  |  |  |  |  |  |  |  |
| **83** |  |  |  |  |  |  |  |  |  |
| **88** |  |  |  |  |  |  |  |  |  |

**(B)**

| **ST** | **Acne** | **Ophthalmic** | **Medical device** | **Soft tissue** | **Dental** | **Spinal disc** | **Bone** | **Skin wound** | **Skin** |
| --- | --- | --- | --- | --- | --- | --- | --- | --- | --- |
| **Type IA_1_** | | | | | | | | | |
| **1** |  |  |  |  |  |  |  |  |  |
| **3** |  |  |  |  |  |  |  |  |  |
| **4** |  |  |  |  |  |  |  |  |  |
| **8** |  |  |  |  |  |  |  |  |  |
| **9** |  |  |  |  |  |  |  |  |  |
| **10** |  |  |  |  |  |  |  |  |  |
| **11** |  |  |  |  |  |  |  |  |  |
| **13** |  |  |  |  |  |  |  |  |  |
| **14** |  |  |  |  |  |  |  |  |  |
| **15** |  |  |  |  |  |  |  |  |  |
| **16** |  |  |  |  |  |  |  |  |  |
| **17** |  |  |  |  |  |  |  |  |  |
| **18** |  |  |  |  |  |  |  |  |  |
| **19** |  |  |  |  |  |  |  |  |  |
| **20** |  |  |  |  |  |  |  |  |  |
| **21** |  |  |  |  |  |  |  |  |  |
| **29** |  |  |  |  |  |  |  |  |  |
| **31** |  |  |  |  |  |  |  |  |  |
| **34** |  |  |  |  |  |  |  |  |  |
| **35** |  |  |  |  |  |  |  |  |  |
| **37** |  |  |  |  |  |  |  |  |  |
| **38** |  |  |  |  |  |  |  |  |  |
| **39** |  |  |  |  |  |  |  |  |  |
| **40** |  |  |  |  |  |  |  |  |  |
| **41** |  |  |  |  |  |  |  |  |  |
| **43** |  |  |  |  |  |  |  |  |  |
| **44** |  |  |  |  |  |  |  |  |  |
| **45** |  |  |  |  |  |  |  |  |  |
| **46** |  |  |  |  |  |  |  |  |  |
| **47** |  |  |  |  |  |  |  |  |  |
| **48** |  |  |  |  |  |  |  |  |  |
| **49** |  |  |  |  |  |  |  |  |  |
| **50** |  |  |  |  |  |  |  |  |  |
| **52** |  |  |  |  |  |  |  |  |  |
| **54** |  |  |  |  |  |  |  |  |  |
| **55** |  |  |  |  |  |  |  |  |  |
| **80** |  |  |  |  |  |  |  |  |  |
| **82** |  |  |  |  |  |  |  |  |  |
| **83** |  |  |  |  |  |  |  |  |  |
| **86** |  |  |  |  |  |  |  |  |  |
| **87** |  |  |  |  |  |  |  |  |  |
| **88** |  |  |  |  |  |  |  |  |  |
| **Type IA_2_** | | | | | | | | | |
| **2** |  |  |  |  |  |  |  |  |  |
| **22** |  |  |  |  |  |  |  |  |  |
| **23** |  |  |  |  |  |  |  |  |  |
| **24** |  |  |  |  |  |  |  |  |  |
| **36** |  |  |  |  |  |  |  |  |  |
| **57** |  |  |  |  |  |  |  |  |  |
| **91** |  |  |  |  |  |  |  |  |  |
| **Type IB** | | | | | | | | | |
| **5** |  |  |  |  |  |  |  |  |  |
| **12** |  |  |  |  |  |  |  |  |  |
| **42** |  |  |  |  |  |  |  |  |  |
| **51** |  |  |  |  |  |  |  |  |  |
| **53** |  |  |  |  |  |  |  |  |  |
| **56** |  |  |  |  |  |  |  |  |  |
| **78** |  |  |  |  |  |  |  |  |  |
| **84** |  |  |  |  |  |  |  |  |  |
| **89** |  |  |  |  |  |  |  |  |  |
| **Type IC** | | | | | | | | | |
| **70** |  |  |  |  |  |  |  |  |  |
| **85** |  |  |  |  |  |  |  |  |  |
| **Type II** | | | | | | | | | |
| **6** |  |  |  |  |  |  |  |  |  |
| **7** |  |  |  |  |  |  |  |  |  |
| **25** |  |  |  |  |  |  |  |  |  |
| **26** |  |  |  |  |  |  |  |  |  |
| **27** |  |  |  |  |  |  |  |  |  |
| **28** |  |  |  |  |  |  |  |  |  |
| **30** |  |  |  |  |  |  |  |  |  |
| **58** |  |  |  |  |  |  |  |  |  |
| **59** |  |  |  |  |  |  |  |  |  |
| **60** |  |  |  |  |  |  |  |  |  |
| **61** |  |  |  |  |  |  |  |  |  |
| **62** |  |  |  |  |  |  |  |  |  |
| **63** |  |  |  |  |  |  |  |  |  |
| **64** |  |  |  |  |  |  |  |  |  |
| **65** |  |  |  |  |  |  |  |  |  |
| **66** |  |  |  |  |  |  |  |  |  |
| **67** |  |  |  |  |  |  |  |  |  |
| **68** |  |  |  |  |  |  |  |  |  |
| **69** |  |  |  |  |  |  |  |  |  |
| **71** |  |  |  |  |  |  |  |  |  |
| **72** |  |  |  |  |  |  |  |  |  |
| **79** |  |  |  |  |  |  |  |  |  |
| **Type III** | | | | | | | | | |
| **32** |  |  |  |  |  |  |  |  |  |
| **33** |  |  |  |  |  |  |  |  |  |
| **73** |  |  |  |  |  |  |  |  |  |
| **74** |  |  |  |  |  |  |  |  |  |
| **75** |  |  |  |  |  |  |  |  |  |
| **76** |  |  |  |  |  |  |  |  |  |
| **77** |  |  |  |  |  |  |  |  |  |
| **81** |  |  |  |  |  |  |  |  |  |
| **90** |  |  |  |  |  |  |  |  |  |
