# Supplementary material for: An Expanded Multilocus Sequence Typing Scheme for Propionibacterium acnes: Investigation of ‘Pathogenic’, ‘Commensal’ and Antibiotic Resistant Strains
Source: PLoS One. 2012 Jul 30;7(7):e41480. doi: 10.1371/journal.pone.0041480 (PMC3408437; doi:10.1371/journal.pone.0041480)
Supplement: Table S2 — Comparison of P. acnes MLST schemes. The eMLST (eight loci) and Aarhus MLST (nine loci) schemes were compared against a panel of 86 isolates representing different phylogenetic groups of P. acnes (IA1, IA2, IB, IC, II). STs were clustered within the same CC if they shared 7/8 alleles (eMLST) or 8/9 alleles (Aarhus) with at least one other ST. (DOC) [file pone.0041480.s007.doc]

|  |  |  |  | **eMLST (Belfast)** | | | **Aarhus** | |
| --- | --- | --- | --- | --- | --- | --- | --- | --- |
| **Isolate** | **Region** | **Source** | ***recA* type** | **Allelic Profile** | **ST** | **CC**^a^ | **ST** | **CC**^a^ |
| **Type IA_1_** |  |  |  |  |  |  |  |  |
| NCTC737 | UK | Acne | IA | 1-1-1-3-1-1-1-1 | 1 | 1 | 18 | 18 |
| HL072PA2 | USA | Acne | IA | 1-1-1-3-1-1-1-1 | 1 | 1 | 18 | 18 |
| HL072PA1 | USA | Acne | IA | 1-1-1-3-1-1-1-1 | 1 | 1 | 18 | 18 |
| J165 | USA | Skin | IA | 1-1-1-3-1-1-1-1 | 1 | 1 | 18 | 18 |
| HL013PA2 | USA | Acne | IA | 1-1-1-3-1-1-1-1 | 1 | 1 | 18 | 18 |
| HL087PA2 | USA | Acne | IA | 1-1-1-3-1-1-1-1 | 1 | 1 | 18 | 18 |
| HL063PA1 | USA | Skin | IA | 1-1-1-3-1-1-1-1 | 1 | 1 | 18 | 18 |
| HL027PA2 | USA | Skin | IA | 1-1-1-3-1-1-1-1 | 1 | 1 | 12 | 18 |
| HL002PA3 | USA | Acne | IA | 1-1-1-3-1-1-1-1 | 1 | 1 | 59 | 18 |
| HL002PA2 | USA | Acne | IA | 1-1-1-3-1-1-1-1 | 1 | 1 | 59 | 18 |
| HL046PA2 | USA | Acne | IA | 1-1-1-3-1-1-1-1 | 1 | 1 | 59 | 18 |
| HL036PA3 | USA | Skin | IA | 1-1-1-3-1-1-1-1 | 1 | 1 | 8 | 18 |
| HL036PA2 | USA | Skin | IA | 1-1-1-3-1-1-1-1 | 1 | 1 | 8 | 18 |
| HL036PA1 | USA | Skin | IA | 1-1-1-3-1-1-1-1 | 1 | 1 | 8 | 18 |
| 15.2.L1 | Denmark | [21] | IA | 1-1-1-3-1-1-1-1 | 1 | 1 | 29 | 18 |
| 27.1.R1 | Denmark | [21] | IA | 1-1-1-3-1-1-1-1 | 1 | 1 | 29 | 18 |
| HL096PA3 | USA | Skin | IA | 1-1-1-3-1-1-3-1 | 8 | 1 | 18 | 18 |
| HL020PA1 | USA | Acne | IA | 1-1-1-3-1-1-4-1 | 9 | 1 | 77 | 18 |
| HL005PA2 | USA | Skin | IA | 1-1-10-3-1-1-6-1 | 15 | 1 | 12 | 18 |
| HL005PA3 | USA | Skin | IA | 1-1-10-3-1-1-1-1 | 16 | 1 | 12 | 18 |
| 266 | France | Pulmonary | IA | 5-1-1-3-1-1-1-1 | 20 | 1 | 18 | 18 |
| HL099PA1 | USA | Acne | IA | 1-1-1-3-1-1-2-2 | 3 | 3 | 3 | 3 |
| HL056PA1 | USA | Skin | IA | 1-1-1-3-1-1-2-2 | 3 | 3 | 3 | 3 |
| HL096PA2 | USA | Skin | IA | 1-1-1-3-1-1-2-2 | 3 | 3 | 3 | 3 |
| HL053PA1 | USA | Acne | IA | 1-1-1-3-1-1-2-2 | 3 | 3 | 3 | 3 |
| HL074PA1 | USA | Skin | IA | 1-1-1-3-1-1-2-2 | 3 | 3 | 3 | 3 |
| HL007PA1 | USA | Skin | IA | 1-1-1-3-1-1-2-2 | 3 | 3 | 3 | 3 |
| HL043PA1 | USA | Acne | IA | 1-1-1-3-1-1-2-2 | 3 | 3 | 58 | 3 |
| HL043PA2 | USA | Acne | IA | 1-1-1-3-1-1-2-2 | 3 | 3 | 58 | 3 |
| HL083PA1 | USA | Acne | IA | 1-1-1-3-1-1-2-2 | 3 | 3 | 3 | 3 |
| HL005PA1 | USA | Skin | IA | 1-1-1-3-1-1-5-2 | 11 | 3 | 3 | 3 |
| HL038PA1 | USA | Acne | IA | 1-1-1-3-1-1-2-4 | 10 | 3 | 3 | 3 |
| HL078PA1^b^ | USA | Skin | IA | 1-1-1-3-1-14-2-3 | 14 | 3 | 63 | S |
| HL045PA1 | USA | Acne | IA | 1-10-1-3-1-1-2-2 | 17 | 3 | 3 | 3 |
| SK137 | USA | Skin | IA | 8-1-1-3-1-1-2-5 | 18 | 3 | 3 | 3 |
| HL025PA1 | USA | Skin | IB | 1-1-1-3-1-1-8-6 | 4 | 4 | 27 | 28 |
| 3.4.L2 | Denmark | [21] | IB | 1-1-1-3-1-1-8-6 | 4 | 4 | 27 | 28 |
| 1.5.L1 | Denmark | [21] | IB | 1-1-1-3-1-1-8-6 | 4 | 4 | 27 | 28 |
| 30.2.L1 | Denmark | [21] | IB | 1-1-1-3-1-1-8-6 | 4 | 4 | 27 | 28 |
| 24.1.A1 | Denmark | [21] | IB | 1-1-1-3-1-1-8-6 | 4 | 4 | 27 | 28 |
| HL086PA1 | USA | Skin | IB | 1-1-1-3-1-1-8-6 | 4 | 4 | 31 | 31 |
| HL092PA1 | USA | Acne | IB | 1-1-1-3-1-1-8-6 | 4 | 4 | 31 | 31 |
| HL110PA1 | USA | Acne | IB | 1-1-1-3-1-1-8-6 | 4 | 4 | 31 | 31 |
| HL053PA2 | USA | Acne | IB | 1-1-1-3-1-1-8-6 | 4 | 4 | 31 | 31 |
| 16.2.R1 | Denmark | [21] | IB | 1-1-1-3-1-1-8-6 | 4 | 4 | 32 | 31 |
| HL082PA1 | USA | Acne | IB | 1-1-1-3-1-1-7-6 | 13 | 4 | 31 | 31 |
| HL110PA2 | USA | Acne | IB | 1-1-1-1-1-1-8-6 | 21 | 4 | 31 | 31 |
| 39.1.A1 | Denmark | [21] | IB | 1-1-1-1-1-1-8-6 | 21 | 4 | 31 | 31 |
| 6.1.A1 | Denmark | [21] | IB | 1-1-1-1-1-1-8-6 | 21 | 4 | 31 | 31 |
| 15.2.R1 | Denmark | [21] | IB | 1-1-1-1-1-1-8-22 | 34 | 4 | 31 | 31 |
| SK187 | USA | Skin | IB | 16-1-1-15-1-4-8-6 | 19 | S | 67 | 28 |
| **Type IA_2_** |  |  |  |  |  |  |  |  |
| HL037PA1 | USA | Skin | IB | 1-1-1-5-1-4-8-2 | 2 | 2 | 67 | 28 |
| HL025PA2 | USA | Skin | IB | 1-1-1-5-1-4-8-2 | 2 | 2 | 65 | 28 |
| HL059PA2^c^ | USA | Skin | IB | 1-1-1-5-1-4-8-2 | 2 | 2 | 28 | 28 |
| HL059PA1 | USA | Skin | IB | 1-1-1-5-1-4-8-2 | 2 | 2 | 28 | 28 |
| HL005PA4 | USA | Skin | IB | 1-1-1-5-1-4-8-2 | 2 | 2 | 28 | 28 |
| HL002PA1 | USA | Acne | IB | 1-1-1-5-1-4-8-2 | 2 | 2 | 28 | 28 |
| HL027PA1 | USA | Skin | IB | 1-1-1-5-1-4-8-2 | 2 | 2 | 28 | 28 |
| HL083PA2 | USA | Acne | IB | 1-1-1-5-1-4-8-2 | 2 | 2 | 28 | 28 |
| HL046PA1 | USA | Acne | IB | 1-1-1-5-1-4-8-2 | 2 | 2 | 28 | 28 |
| HL013PA1 | USA | Acne | IB | 1-1-1-5-1-4-8-2 | 2 | 2 | 28 | 28 |
| HL050PA3 | USA | Skin | IB | 1-1-1-5-1-4-8-2 | 2 | 2 | 28 | 28 |
| HL087PA1 | USA | Acne | IB | 1-1-1-5-1-4-8-2 | 2 | 2 | 28 | 28 |
| HL087PA3 | USA | Acne | IB | 1-1-1-5-1-4-8-2 | 2 | 2 | 28 | 28 |
| HL067PA1 | USA | Acne | IB | 1-1-1-5-1-4-8-9 | 24 | 2 | 64 | 28 |
| 25.1.L1 | Denmark | [21] | IB | 1-1-1-18-1-4-8-2 | 57 | 2 | 28 | 28 |
| HL050PA1 | USA | Skin | IB | 1-1-1-5-1-4-9-2 | 91 | S | 28 | 28 |
| HL030PA2 | USA | Skin | IB | 1-1-1-5-3-5-8-7 | 22 | S | 66 | 28 |
| 20.2.R1 | Denmark | [21] | IB | 1-1-1-5-3-5-8-7 | 22 | S | 30 | 28 |
| HL063PA2 | USA | Skin | IB | 1-18-1-5-3-5-8-8 | 23 | S | 70 | 28 |
| **Atypical (IC)** |  |  |  |  |  |  |  |  |
| HL097PA1 | USA | Acne | IA | 9-1-4-8-6-8-14-14 | 70 | S | 74 | S |
| **Type IB** |  |  |  |  |  |  |  |  |
| HL030PA1 | USA | Acne | IB | 1-1-1-4-1-4-8-6 | 5 | 5 | 36 | 36 |
| KPA171202^c^ | Germany | Plate | IB | 1-1-1-4-1-4-8-6 | 5 | 5 | 36 | 36 |
| 6609 | Hungary | Skin | IB | 1-1-1-4-1-4-8-6 | 5 | 5 | 36 | 36 |
| 36.1.A1 | Denmark | [21] | IB | 1-1-1-4-1-4-8-6 | 5 | 5 | 42 | 36 |
| CCUG32901 | Sweden | Blood | IB | 1-1-1-4-1-4-8-6 | 5 | 5 | 33 | 36 |
| 21.1.L1 | Denmark | [21] | IB | 1-1-12-4-1-4-8-6 | 56 | 5 | 36 | 36 |
| **Type II** |  |  |  |  |  |  |  |  |
| HL082PA2 | USA | Acne | II | 17-4-2-4-2-3-10-10 | 6 | 6 | 62 | 60 |
| HL060PA1 | USA | Acne | II | 17-4-2-4-2-3-10-10 | 6 | 6 | 69 | 60 |
| HL110PA3^c^ | USA | Acne | II | 15-4-2-4-2-3-10-10 | 7 | 6 | 73 | 60 |
| HL110PA4 | USA | Acne | II | 15-4-2-4-2-3-10-10 | 7 | 6 | 73 | 60 |
| HL103PA1^c^ | USA | Acne | II | 17-9-2-4-2-3-10-10 | 25 | 6 | 60 | 60 |
| HL050PA2 | USA | Skin | II | 17-4-2-17-2-3-11-11 | 26 | S | 60 | 60 |
| J139^c^ | USA | Skin | II | 17-4-2-16-2-12-10-12 | 28 | 72 | 73 | 60 |
| HL001PA1 | USA | Skin | II | 17-4-2-4-2-6-10-12 | 30 | 72 | 60 | 60 |
| ATCC11828 | USA | Abscess | II | 17-4-2-4-9-12-10-13 | 27 | S | N | 60 |
| **Total** | - | - | - | - | 33 | 7 (7) | 28 | 6 (2) |

^a^S=singleton;

^b^HL078PA1 was incorrectly assigned as ST6 by Kilian et al. [18] using our previous seven gene MLST scheme and database (correct ST = 57; McDowell et al [17]);

^c^HL059PA2, KPA171202, HL110PA3, HL103PA1, J139 were previously assigned as ST68, ST34, ST75, ST72 and ST78, respectively by Kilian et al. [18] using the Aarhus MLST approach. Based on our analyses of the same sequence data, these designations are also incorrect. The correct ST assignments for these isolates are as listed.
